# Expression of the Gene for Autotransporter AutB of *Neisseria meningitidis* Affects Biofilm Formation and Epithelial Transmigration

**DOI:** 10.3389/fcimb.2016.00162

**Published:** 2016-11-22

**Authors:** Jesús Arenas, Fernanda L. Paganelli, Patricia Rodríguez-Castaño, Sara Cano-Crespo, Arie van der Ende, Jos P. M. van Putten, Jan Tommassen

**Affiliations:** ^1^Department of Molecular Microbiology and Institute of Biomembranes, Utrecht UniversityUtrecht, Netherlands; ^2^Department of Medical Microbiology, University Medical Center UtrechtUtrecht, Netherlands; ^3^Department of Medical Microbiology, Academic Medical CenterAmsterdam, Netherlands; ^4^Department of Infectious Diseases and Immunology, Faculty of Veterinary Medicine, Utrecht UniversityUtrecht, Netherlands

**Keywords:** autotransporters, protein secretion, biofilms, infection, *Neisseria meningitidis*, *Haemophilus influenzae*, pathogenesis

## Abstract

*Neisseria meningitidis* is a Gram-negative bacterium that resides as a commensal in the upper respiratory tract of humans, but occasionally, it invades the host and causes sepsis and/or meningitis. The bacterium can produce eight autotransporters, seven of which have been studied to some detail. The remaining one, AutB, has not been characterized yet. Here, we show that the *autB* gene is broadly distributed among pathogenic *Neisseria* spp. The gene is intact in most meningococcal strains. However, its expression is prone to phase variation due to slipped-strand mispairing at AAGC repeats located within the DNA encoding the signal sequence and is switched off in the vast majority of these strains. Moreover, various genetic disruptions prevent *autB* expression in most of the strains in which the gene is in phase indicating a strong selection against AutB synthesis. We observed that *autB* is expressed in two of the strains examined and that AutB is secreted and exposed at the cell surface. Functionality assays revealed that AutB synthesis promotes biofilm formation and delays the passage of epithelial cell layers in *vitro*. We hypothesize that this autotransporter is produced during the colonization process only in specific niches to facilitate microcolony formation, but its synthesis is switched off probably to evade the immune system and facilitate human tissue invasion.

## Introduction

The Gram-negative diplococcus *Neisseria meningitidis* is a common inhabitant of the human nasopharynx, but it is also the causative agent of meningococcal disease, a life-threatening infection characterized by fast development of septicemia and/or meningitis. The colonization and its persistence in the host involve the formation of microcolonies in the nasopharynx (Sim et al., 2000). Microcolonies are biofilm-like structures, which are defined as multicellular microbial communities often encased within a self-produced extracellular matrix (Costerton et al., 1995) and presumably help the bacteria to survive adverse circumstances, such as host defense mechanisms. Amongst others, several autotransporters (ATs) have been demonstrated to play a role in biofilm formation in *N. meningitidis* (Arenas and Tommassen, 2016).

ATs are a class of proteins secreted by Gram-negative bacteria (Grijpstra et al., 2013). They contain an N-terminal signal sequence for transport across the inner membrane via the Sec machinery and a C-terminal translocator domain that inserts as a β-barrel in the outer membrane via the Bam complex and that assists in the translocation of an associated passenger domain across the outer membrane. Based on the structure of the translocator domain, two main types of ATs can be discriminated, the classical monomeric ATs and the trimeric ATs. In classical monomeric ATs, the C-terminal translocator domain forms a 12-stranded β-barrel, whilst in trimeric ATs, the translocator domain of each subunit contributes four β-strands to form a similar 12-stranded β-barrel as in the monomeric ATs. The passenger of classical ATs usually forms an extended β-helix on which smaller globular domains are displayed. The C-terminal part of these passengers often harbors a linker domain that, in some cases, has been demonstrated to contain autochaperone activity, i.e., it helps in the folding of the passenger after its secretion to the cell surface (Oliver et al., 2003; Peterson et al., 2010). After secretion, the passenger can remain attached to the cell surface or it can be released into the external medium by one of a variety of possible proteolytic mechanisms. Their functions can be very diverse, but they are often involved in virulence (Grijpstra et al., 2013).

Based on the analysis of genome sequences, it appears that *N. meningitidis* can produce up to eight different ATs, i.e., IgA1 protease, App, AusI, NalP, NhhA, NadA, AutA, and AutB (van Ulsen and Tommassen, 2006). Seven of them have been characterized to at least some extent. The passenger of IgA1 protease consists of two domains, the protease domain, which cleaves, amongst others, human immunoglobulin A1, the IgA subclass that predominates in the nasopharynx, and an α-peptide that can be released from the cell surface together with the protease domain, or it can remain attached to the cell surface where it is implicated in biofilm formation by binding extracellular DNA (eDNA) (Arenas et al., 2013a). Like IgA1 protease, App and AusI (a.k.a. MspA) are monomeric ATs with a protease domain, and both have been implicated in adhesion to epithelial cells (Serruto et al., 2003; Turner et al., 2006). Also NalP has protease activity; amongst others, it releases proteins from the bacterial cell surface, including the α-peptide of IgA1 protease and the neisserial heparin-binding antigen NHBA, a surface-exposed lipoprotein, which, like the α-peptide, is involved in biofilm formation by binding eDNA (van Ulsen et al., 2003; Serruto et al., 2010; Arenas et al., 2013a). Thus, NalP regulates biofilm formation. NhhA and NadA are cell-associated trimeric ATs with a role in adhesion to eukaryotic cells (Capecchi et al., 2005; Scarselli et al., 2006), whilst AutA is a monomeric AT involved in bacterial autoaggregation, a function that affects biofilm structure (Arenas et al., 2015). The remaining AT, AutB, has not been characterized yet. It is evolutionary related to a poorly characterized AT designated Lav, present in some strains of *Haemophilus influenzae*, another inhabitant of the nasopharynx, from which it was suggested to be acquired by horizontal gene transfer. Expression of its gene and its function remain enigmatic.

The expression of the genes for most ATs in *N. meningitidis* is prone to phase variation by slipped-strand mispairing at short nucleotide repeats (Turner et al., 2002; van Ulsen et al., 2006; Arenas et al., 2015). Besides, expression of several AT genes, e.g., *ausI* and *autA*, can be prevented in certain lineages by genetic disruptions, such as deletions or frame-shift mutations, possibly evolved by selection pressure imposed by the immune system (van Ulsen et al., 2006; Arenas et al., 2015). The expression of *autB* also seems to be prone to phase variation. The gene contains a variable number of AAGC nucleotide repeats immediately downstream of the start codon (Peak et al., 1999). These repeats are also present in the *autA* gene, where they were shown to be implicated in phase variation (Arenas et al., 2015). However, expression of *autB* has not been demonstrated so far. In fact, early studies suggested that *autB* is a pseudogene (Ait-Tahar et al., 2000). This conclusion was based on the lack of reactivity of two anti-AutB antisera against nine meningococcal and one gonococcal strains. However, the number of AAGC repeats and the presence of genetic disruptions in the *autB* genes of those strains were not evaluated. Therefore, the conclusion that *autB* is a pseudogene may be premature. The objective of the present work was to determine whether AutB can be produced in *N. meningitidis* and to elucidate its possible function.

## Materials and methods

### Bioinformatics analysis

Amino-acid sequences encoded by the *autB* genes were predicted after alteration of the number of AAGC repeat units to create the correct reading frame and, in some cases, by removal of frameshift mutations and premature stop codons. The cleavage site of the N-terminal signal sequence was predicted using publicly available web tools (http://www.cbs.dtu.dk/services/SignalP/). The mature protein was then used for phylogenetic analysis using the neighbor-joining with bootstrap analysis (100 replications) considering distances based on p-distance with the available MEGA software version 6.0 (http://www.megasoftware.net/) (Tamura et al., 2013). For secondary and tertiary structure predictions, the public web-based programs PsiPred (Buchan et al., 2013) and PHYRE2 (http://www.sbg.bio.ic.ac.uk/phyre2/html/page.cgi?id=index), respectively, were used. Alignment of protein sequences was performed in MAFFT version 7 (http://mafft.cbrc.jp/alignment/server/). AutB variants were defined based on phylogeny of the N-terminal part of the passenger. Each variant was considered when the bootstrap value of the precedent node was 100, the distance within the group was lower than the overall distance for AutB, and the distance from other groups higher than the overall distance for AutB.

### Bacterial strains and growth conditions

Meningococcal strains used in this study include reference strains MC58 (Tettelin et al., 2000), FAM18 (Bentley et al., 2007), α14 (Schoen et al., 2008), and α153 (Schoen et al., 2008). HB-1 (Bos and Tommassen, 2005) and BB-1 (Arenas et al., 2013a) are unencapsulated derivatives of H44/76 and B16B6, respectively. A panel of 102 meningococcal strains isolated from patients suffering from meningococcal disease in the Netherlands was already described (Arenas et al., 2015). All meningococcal strains were grown at 37°C on GC medium base (Difco) supplemented with IsovitaleX (Becton Dickinson) at 37°C in a candle jar overnight. To grow the bacteria in liquid cultures, bacteria were collected from GC plates and diluted in tryptic soy broth (TSB) (Beckton Dickinson) to an OD_550_ of 0.1 and incubated in 25-cm^2^ polystyrene cell culture flasks or 125-ml square media bottles with constant shaking at 110 rpm. *Escherichia coli* strains used in this study were DH5α and BL21(DE3) (Invitrogen), which were grown in Lysogeny broth (LB) or LB agar at 37°C.

For all strains, media were supplemented with the antibiotics kanamycin (100 μg ml^−1^), chloramphenicol (25 μg ml^−1^) or ampicillin (100 μg ml^−1^) when required and with 0.1 mM of isopropyl-β-D-1-thiogalactopyranoside (IPTG) to induce gene expression.

### PCR analysis and sequencing of the *autB* gene

Segments of *autB* were amplified by PCR from bacteria grown on plates with primers listed in Table S1 using High Fidelity Polymerase (Roche Diagnostics GmbH, Germany) or DreamTaq-DNA Polymerase (Fermentas, UK). PCR products were visualized in agarose gels stained with ethidium bromide. When required, they were purified with the PCR Clean-Up System (Promega Corporation) and sequenced at the Macrogen sequencing service (Amsterdam). Large sequences were assembled using the SeqMan II software (DNAstart) using at least two independent PCR reactions.

### Cloning and transformation

For cloning, PCR fragments were obtained from DNA of strain HB-1 using primers described in Table S1. PCR fragments were purified and digested with restriction enzymes (Fermentas, UK) for which sites were included in the primers, to be subsequently cloned in appropriate vectors. To generate an *autB* knockout construct, PCR fragments from immediately upstream and downstream of the *autB* gene were cloned into pKO*nhbA-kan* (Arenas et al., 2013a). In the resulting plasmid, called pKO*autB*-*kan*, these segments flank a kanamycin-resistance cassette.

To generate a plasmid for the production of a part of the passenger domain of AutB of strain HB-1 in *E. coli*, the corresponding PCR product was cloned into pET16b (Invitrogen) resulting in plasmid pET16b-AutBp. To generate plasmids for expression of AutB variants in *N. meningitidis*, the entire *autB* genes from isolates 2081107 and α153 were amplified and cloned into plasmid pFPIORF_1_ (Arenas et al., 2013b), resulting in pENAutB_1_ and pENAutB_2_, respectively. *N. meningitidis* and *E. coli* were transformed with intact or linearized plasmids following standard protocols (Arenas et al., 2015) and transformants were selected on solid medium supplemented with appropriate antibiotics. The proper generation of knockout mutants or the presence of the plasmid was verified by PCR. In addition, protein synthesis was confirmed by Western blotting or gene expression was confirmed by RT-PCR as described below.

### Purification of recombinant AutB and antiserum production

A fragment of the AutB passenger domain of strain HB-1 was purified as described (Arenas et al., 2015). Briefly, the recombinant polypeptide, corresponding to amino-acid residues 151–278 of the mature AutB protein, with an N-terminal His-tag was produced in *E. coli* BL21(DE3) carrying pET16b-AutBp after induction with IPTG and purified as inclusion bodies. The protein band corresponding to the recombinant protein was excised from SDS-polyacrylamide gel electrophoresis (SDS-PAGE) gels and used for the production of rabbit antiserum at Eurogentec (Liège, Belgium). The concentration of the purified protein was determined with the BCA assay kit (Thermo Fisher Scientific, Rockford, IL, USA) and its purity was estimated in SDS-PAGE gels.

### RNA purification and RT-PCR assays

To obtain RNA, cells from exponentially growing cultures were collected by centrifugation for 10 min at 5000 rpm in an Eppendorf Centrifuge 5424, adjusted to an OD_550_ of 4, and resuspended in trizol (Invitrogen, U.K.). Then, 200 μl of chloroform were added per ml of trizol, followed by centrifugation at 5000 rpm for 30 min. The resulting upper layer was mixed with an equal amount of ice-cold 75% ethanol. Next, RNA was isolated using the Nucleospin RNA II kit (Macherey-Nagel, U.S.A.) according to the manufacturer's instructions. The resulting solution was treated with Turbo DNA free (Ambion, Germany) for 1 h at 37°C to remove genomic DNA followed by inactivation of the DNase according to the recommendations of the manufacturer. The resulting pure RNA was used immediately to generate cDNA using the Transcriptor High Fidelity cDNA Synthesis Kit (Roche, The Netherlands). RNA, cDNA, and chromosomal DNA were used as templates in PCRs to determine the generation of specific transcripts with primers listed in Table S1. PCRs started with an incubation for 5 min at 95°C, followed by 30 cycles of 30 s at 95°C, 30 s at 65°C and 10 s at 72°C. Reactions ended with 10 min of incubation at 72°C.

### SDS-PAGE and Western Blotting

Whole cell lysates, supernatants and cell envelopes were prepared and adjusted as previously described (Arenas et al., 2015). Protein concentrations were determined with the BCA assay kit. Before SDS-PAGE, samples were diluted 1:1 in double-strength sample buffer and heated for 10 min at 100°C. Proteins separated on gels were stained with Coomassie brilliant blue G250 or transferred to nitrocellulose membranes. These membranes were next blocked with phosphate-buffered saline (PBS) containing 0.1% (v/v) Tween 20 and 0.5% (w/v) non-fat dried milk (PBS-T-M), and then incubated with primary antibodies and subsequently with horseradish peroxidase-conjugated goat anti-rabbit IgG or anti-mouse IgG antibodies (Biosource International), as previously described (Arenas et al., 2006). All incubations were performed for 1 h and followed by three washes for 15 min with PBS-T-M. Blots were developed with the Pierce ECL Western Blotting Substrate. The monoclonal antibody MN2D6D directed against RmpM and the antiserum directed against fHbp were generously provided by the Netherlands Vaccine Institute (Bilthoven, The Netherlands) and by GlaxoSmithKline (Rixensart, Belgium), respectively. The antisera directed against the α-peptide and the translocator domain of IgA protease were from our laboratory collection (Roussel-Jazédé et al., 2014).

### Proteinase K accessibility assays

Proteinase K accessibility assays were performed as described (Arenas et al., 2015) with few modifications. Briefly, bacteria recovered from a culture grown for 4 h in TSB were adjusted to an optical density at 550 nm (OD_550_) of 1 and incubated with 2 μg ml^−1^ of proteinase K (Fermentas) for 1 h at 37°C, after which the protease was inactivated with 2 mM phenylmethylsulfonyl fluoride Sigma-Aldrich). Cells were harvested by centrifugation and protein degradation was examined by SDS-PAGE and Western blotting.

### Settling experiments and biofilm formation

For biofilm formation and settling experiments, bacteria were initially grown in TSB with or without IPTG and adjusted to a similar OD_550_. Settling experiments were performed as described (Arenas et al., 2015). Biofilm formation was analyzed under static conditions in polystyrene plates and in a flow-cell model as described before (Arenas et al., 2013a, 2015), with some modifications. Where indicated, 100 μg ml^−1^ of DNase I was added to the cultures during biofilm formation. For the flow-cell model, pictures were taken at different time intervals during biofilm formation. After 16 h, biofilms were stained with LIVE/DEAD stain (Life Technologies Europe BV, the Netherlands) dissolved in Dulbecco's PBS (DPBS) (Lonza, USA) as suggested by the manufacturer. Structural parameters of the biofilms were analyzed using COMSTAT (Heydorn et al., 2000)/MATLAB R2010b software (The MathWorks) program, using eight image stacks randomly generated of each sample. For statistical analysis, data from at least three independent experiments performed in duplicate were used. Statistical comparisons of all biofilm parameters were calculated using an unpaired *t*-test with GRAPH PAD v 6.0 (Graph Pad Software, Inc).

### Infection assays

The epithelial cell line NCI-H292 (ATCC CRL 1848) was cultured in RPMI 1640 medium supplemented with 5% fetal calf serum, which was heat inactivated before use (1 h at 56°C). All cell-culture medium components were purchased from Laboratories PAA. The cells were cultured at 37°C in a humidified atmosphere containing 5% CO_2_ and maintained in 25-cm^2^ tissue-culture flasks (Nunc) until ~80% confluence was reached.

Bacterial adherence was determined as previously described (van Putten and Paul, 1995). In short, 2 days before the assays, NCI-H292 cells from 4 to 25 passages were seeded in 24-well plates. Bacteria grown overnight on plate were suspended in HEPES buffer (10 mM HEPES, 145 mM NaCl, 5 mM KCl, 5 mM glucose, 1 mM CaCl_2_ and 1 mM MgCl_2_, pH 7.2), washed by centrifugation (1500 *g*, 10 min) and incubated with cultured cells at a multiplicity of infection of 100 for 3 h. Non-adherent bacteria were removed by sequential washings with DPBS, and the viable adhering bacteria were counted by determining the colony-forming units (CFU) after disruption of the cell layer with 1% saponin diluted in DPBS for 15 min and subsequent mechanic homogenization.

Passage of cell layers was assayed as described previously (van Schilfgaarde et al., 1995) with modifications. In short, cells were grown on transparent tissue culture inserts with 1-μm pores (model no. 353104; Falcon) placed in 24-well plates (Falcon) containing 0.5 ml of medium. The culture medium was refreshed every 2 days. After 6 days, the transepithelial resistance of the cellular layers was measured with the Millicell-ERS Resistance system (Millipore, Bedford, Mass.), and the insert was moved to a new well containing 300 μl of RPMI in the basal compartment with 100 μg/ml of DNase I for avoid biofilm formation. Before starting the infection, the culture medium above the insert was replaced, and the cellular layers were then infected with ~10^7^ CFU for in total 12 h. After 3 h, the medium in the upper compartment was replaced to avoid medium acidification, and the number of CFU in the basal compartment was determined at regular time intervals.

### Nucleic acid accession numbers

The nucleotide sequences of the *autB* genes of disease isolates 2081107, 2061551, and 2070077 were deposited in GenBank ID (KT367782, KT367783, and KT367784, respectively).

## Results

### Characterization and distribution of the *autB* gene

**Figure 1:**
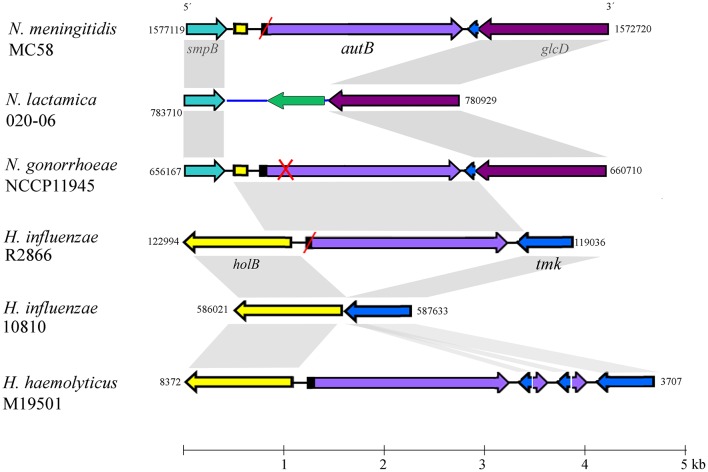
**Genomic context of the ***autB*** gene**. The relevant part of the genome sequences of *N. meningitidis* strain MC58, *N. lactamica* strain 020-06, *N. gonorrhoeae* strain NCCP11945, *H. influenzae* strains R2866 and 10810, and *H. haemolyticus* strain M19501 are schematically depicted. Numbers at the side of each map indicate the first and last nucleotide position of the DNA fragment shown in accordance with genome annotations. The genes (arrows) and intergenic regions (lines) with high sequence similarity between different genome sequences are colored identically. Gray shadowing indicates regions with >85% of sequence identity. Red slashes indicate that the gene is out of phase at the AAGC repeats but can potentially be expressed after phase variation. A red cross indicates that the gene is disrupted and cannot be expressed even if it is in phase at the AAGC repeat region. The genes flanking *autB* are conserved in the *Neisseria* genomes. The upstream gene, *smpB*, encodes a protein of 148 amino-acid residues (aa), which is a component of the *trans*-translation system for releasing stalled ribosomes from damaged messenger RNAs. The downstream gene is a homolog of *glcD* of *E. coli*, which encodes the D subunit of glycolate oxidase. *N. lactamica* misses the *autB* gene and its flanking sequences; instead, it contains an alternative intergenic sequence (colored blue) and a gene encoding a hypothetical protein of 168 aa with a conserved DUF1877 domain (colored green). BLAST searches using this gene as query indicated that it is present in the genomes of other *Neisseria* spp. that lack *autB* as well as in many other bacteria. The *autB* gene is also present in some strains of *Haemophilus* spp. but in a different genomic context. In these species, the gene is located between the *holB* gene, which encodes the δ′ subunit of DNA polymerase III, and the *tmk* gene, which encodes thymidylate kinase. It is noteworthy that the intergenic region upstream of *autB* in the *Neisseria* genomes contains part of the 5′ end of the *holB* gene of *Haemophilus*; also downstream of *autB*, a part of the intergenic region and the 3′ end of the *tmk* gene is shared between *N. meningitidis* and *Haemophilus*, suggesting that a DNA segment containing *autB*, the flanking intergenic regions and parts of the *holB* and *tmk* genes from *Haemophilus* was inserted en bloc into a common ancestor of *N. meningitidis* and *N. gonorrhoeae*. Interestingly, a DNA fragment containing the 5′ ends of *autB* and *tmk* and their intergenic region is repeated several times downstream of *autB* in *H. haemolyticus* strain M19501.

BLASTn searches identified the *autB* gene in available genome sequences of the pathogenic Neisseria spp. *N. meningitidis* and *N. gonorrhoeae* but not in those of non-pathogenic species, such as *N. lactamica* (Table 1 and Table S2). The gene was also identified in some strains of Haemophilus spp. (Table 1 and Table S2). Figure 1 illustrates the position of the *autB* gene in representative genomes. The *autB* gene is ubiquitous in both pathogenic Neisseria spp. with the exception of one meningococcal strain, i.e., strain 053442. In this strain, as well as in *N. lactamica*, the flanking genes are preserved, but the *autB* gene is substituted by a 1415-bp sequence. Interestingly, the sequences flanking the *autB* gene in *H. influenzae* strains, including the 5′ end of the *holB* gene and the 3′ end of the *tmk* gene are also present as intergenic regions flanking *autB* in *N. meningitidis* and *N. gonorrhoeae* (Figure 1). This indicates that *autB* and the flanking regions may have been transferred en bloc from Haemophilus to Neisseria as suggested previously (Davis et al., 2001). However, many Haemophilus strains do neither contain the *autB* gene (Table 1), nor the alternative region found in *N. lactamica* (Figure 1 and data not shown). Interestingly, a sequence containing the 3′ ends of the *autB* and *tmk* genes is tandem repeated in *H. haemolyticus* (Figure 1), indicating that this region is prone to genetic recombination.

**Table 1:** **Distribution of ***autB*** within available genome sequences of ***Neisseria*** and ***Haemophilus*** species**.

**Species**	**Strains analyzed**	**Presence *autB***	**Intact^a^**	**In frame^b^**
*N. meningitidis*	118	117	104	2
*N. gonorrhoeae*	16	16	0	0
*N. lactamica*	4	0		
*N. flavescens*	2	0		
*N. polysaccharea*	2	0		
*N. sicca*	4	0		
*H. influenzae*	15	7	7	2
*H. haemolyticus*	9	2	2	2
*H. parainfluenzae*	2	1	1	?
*H. aegyptius*	3	3	3	0

a *Strains having an intact autB gene that is either in or out of frame at the AAGC repeats*.

b *Strains having an intact autB gene that is in frame because of an appropriate number of AAGC repeats. In the case of the H. parainfluenzae strain, it cannot be determined whether the gene is in frame because the sequence upstreamŏf the AAGC repeats is not available*.

The genetic characteristics of the *autB* gene in a panel of Neisseria and Haemophilus strains are listed in Table S2. Table 1 summarizes the numbers of strains in which the gene can be expressed. The number of AAGC nucleotide repeats is considerably lower in Neisseria spp. (3–19) than in Haemophilus spp. (17–30 repeats). In the meningococcal *autA* gene, the same repeat is present, but the range of copies is considerably larger (6–40) (Arenas et al., 2015) than in meningococcal *autB*. In 12 of the 117 meningococcal strains examined (10.3%), the *autB* gene is in frame at these repeats, but in 10 of them the gene is disrupted further downstream of the repeats by frame-shift mutations, premature stop codons or insertion of a transposase element (Table S2). Thus, only two strains (1.7%), i.e., α153 and LNP27256, putatively express *autB* (Table 1). In stark contrast, in the 105 meningococcal strains, in which the *autB* gene is out of phase at the AAGC repeats, the gene is further intact with only three exceptions (Table S2). Hence, all these strains can potentially express the gene after phase variation occurs, but, apparently, this is prevented by a very strong selection pressure. Due to downstream disruptions, none of the gonococcal strains can express *autB*, independent of number or repeats. Such disruptions are not present in the *autB* genes of the Haemophilus strains examined, and the gene is in frame in 28% of them (Table S2), but all strains can potentially express *autB* after slipped-strand mispairing. To summarize, *autB* can be expressed, but expression seems to be prone to phase variation at the AAGC repeats and to different genetic disruptions. In addition, these data indicate a strong selection against *autB* expression in Neisseria, but not in Haemophilus spp.

### Structure and variability of AutB

The *autB* gene from *N. meningitidis* reference strain MC58 is not expressed because the number of AAGC repeats renders the gene out of frame (Table S2). Deletion of two repeats results in an N-terminally extended ORF coding for a hypothetical protein of 674 amino-acid residues (aa). The signal peptide cleavage site in this protein was predicted between aa residues A_30_ and V_31_ resulting in a mature protein of 644 aa, which is similar to that of AutA (660 aa), and with a calculated molecular mass of 73.2 kDa. The passenger of AutB is constituted of two parts, a C-terminal linker domain (aa 231–338), which shows homology to the autochaperone domain of other ATs and could accordingly be modeled as a right-handed β-helix (data not shown), and an N-terminal domain. Secondary structure predictions indicate that this N-terminal part of the passenger contains two conserved α-helices (with approximate positions between aa 1–20 and 90–110 of the mature aa sequences) separated by a largely unstructured region of ~80 aa and followed by a segment that is rich in β-sheet but occasionally also contains some α-helix prediction (Figure S1). Rather similar secondary structure was predicted for the corresponding fragment of AutA (Figure S1). The passengers of AutB proteins each contain one or two pairs of cysteines, which probably form a disulfide bond, but they are located at different positions in the sequence (Figure S2). The translocator domain of AutB (aa 360–644) is predicted to form a 12-stranded β-barrel that is connected via an α-helix (aa 337–354) to the passenger.

**Figure 2:**
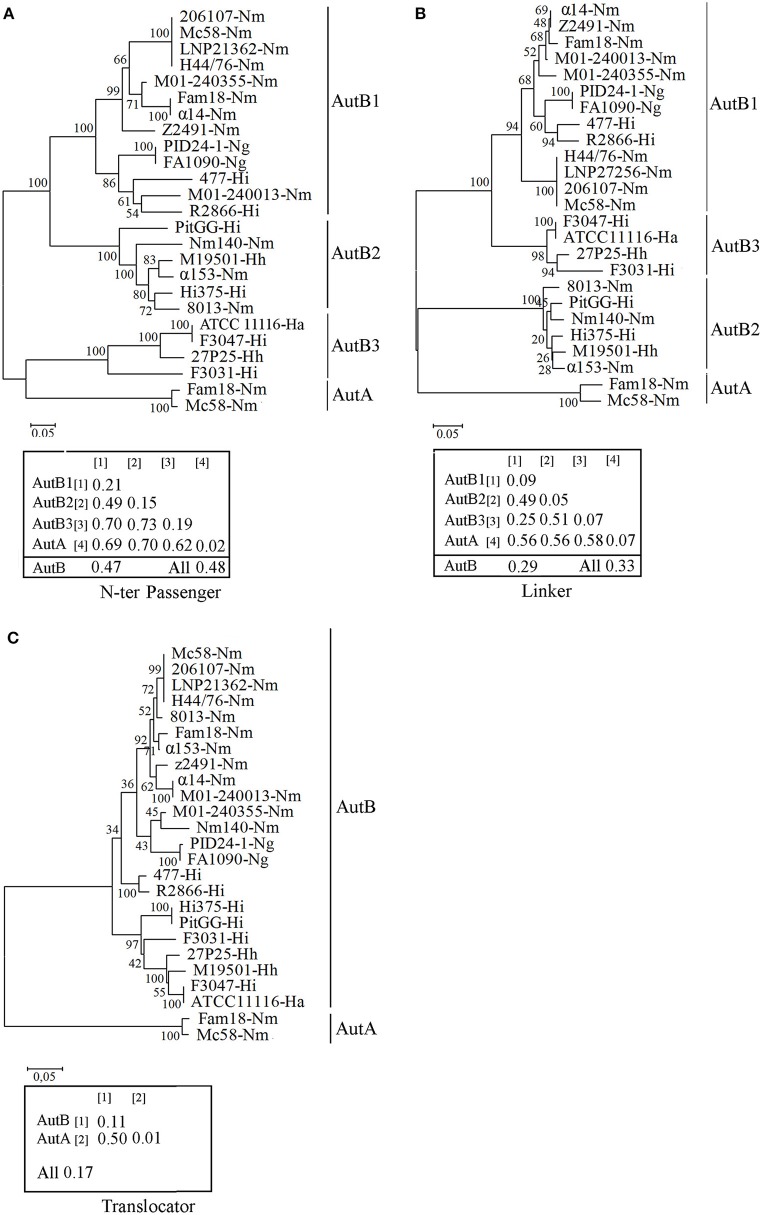
**Phylogenetic trees of ***autB*** gene sequences**. Cluster analysis was performed on the N-terminal part of the passenger **(A)**, the linker **(B)**, and the translocator domain **(C)** of mature AutB and AutA proteins derived from representative genomes of *N. meningitidis* (*Nm), N. gonorrhoeae* (*Ng*), *H. influenzae* (*Hi*), *H. aegyptius* (*Ha*), and *H. haemolyticus (Hh)* strains. The *autB* genes from *H. influenzae* strain R2866 and *H. aegyptius* ATCC11116 were previously called *lav* and *las*, respectively (Davis et al., 2001). The bootstraps values are shown on the branches. Genetic distances within and between groups and overall mean distances for AutBs are included in the insets.

To determine sequence conservation, the predicted mature AutB proteins from several *Neisseria* and *Haemophilus* strains were aligned. The alignments (see Figure S2 for representatives) revealed that the sequence of the translocator domain was better conserved than that of the passenger. The translocator domain also showed considerable similarity to that of AutA of MC58 (not included in the alignments, but see below). However, the passenger domain showed high sequence diversity (see Figure S2 for examples). To investigate this variability in more detail, we performed independent phylogenetic analysis for the N-terminal part of the passenger, the linker and the translocator domain of representative AutB proteins and AutA of MC58 (Figure 2). Indeed, the translocator domain showed limited variability as compared with the other two domains (compare overall mean distances of the separate domains of the AutB proteins). Although similar to the translocator domain of the AutB proteins, the translocator domain of AutA clustered in a different branch, demonstrating that AutA is a different AT, which is consistent with its distinct genomic location. When the N-terminal domain of the passengers was analyzed, three major branches were identified, here designated AutB1, AutB2, and AutB3. The assignment of the AutB proteins in the different strains to these clusters is shown in Table S2. AutA again formed a separate branch. Interestingly, the phylogenic relationships of the AutB proteins do not parallel the boundaries of the species in which these proteins are found, suggesting that specific variants did not evolve in one specific organism, but that there is regular exchange of the genes between these species. AutB1 is the predominant variant in three species, i.e., *N. meningitidis, N. gonorrhoeae*, and *H. influenzae*. The AutB3 variant was found in three *Haemophilus* spp. and the AutB2 variant is present in all species except in *N. gonorrhoeae* and *H. parainfluenzae*. Variant AutB3 showed a larger diversity in the N-terminal domain of the passenger than the other variants and, interestingly, clustered with the passenger of AutA, suggesting the exchange of sequences between both AT, but limited to this domain. Cluster analysis of the linker domain also indicated the presence of the three main branches with AutA being a separate cluster. The groups were constituted of the same *autB* genes as for the N-terminal domain of the passenger; therefore, the same nomenclature is maintained. However, the relation between the groups varied notably. In this part of the protein, AutB1 was closely related to AutB3 and only distantly related to AutB2. This analysis confirmed that the passenger is rather variable as compared with the translocator domain, which is in agreement with its expected surface exposure and accessibility to the immune system. In addition, this analysis reflects shuffling of different domains within the passenger as suggested before (Davis et al., 2001).

To gain more insight in the evolution of *autB*, we calculated the GC content in the three regions in several *autB* genes. The GC content was similar in the regions encoding the N-terminal part of the passenger and the linker domain (35 ± 2%), but remarkably different for that encoding the translocator domain (44 ± 2%), which suggests that the passenger and the translocator domains have different evolutionary origins that are both different from the pathogenic *Neisseria* spp. with a GC content of 52% in their genome sequences. No differences were found in this respect between the three *autB* variants, suggesting that they evolved in the same species. In the *autA* gene, the GC content of the three regions was remarkably different, i.e., 40 ± 3, 52 ± 3, and 56 ± 1% for the N-terminal part of the passenger, the linker and the translocator domain, respectively. The two latter regions are closer to the average GC content in *Neisseria* genomes. Together, this analysis supports the hypothesis that both ATs have a different origin and suggests the exchange of genetic material between genes of different ATs in the region encoding the passenger.

### Phase variation of *autB* in clinical meningococcal isolates

To study phase variation of *autB*, we analyzed large sets of meningococcal disease isolates from the same genetic origin. If phase variation indeed occurs frequently, different isolates of the same cc should contain different numbers of repeats and, in the absence of selection pressure, ~33% should have the gene in the proper reading frame. We analyzed collections of cc213 and cc32 isolates, two hyper-invasive clonal complexes. The number of repeat units in the *autA* gene of these isolates was previously analyzed (Arenas et al., 2015) allowing us to compare phase variation in these two AT genes. In cc213 strain M01-240355, *autB* is in phase at the AAGC repeat but it is disrupted by a downstream frameshift mutation (Table S2). In the cc32 strains listed in Table S2 (*n* = 17), the *autB* gene is out of phase but it does not contain additional disruptions. All these genomes harbor an AutB1 variant (Table S2).

**Figure 3:**
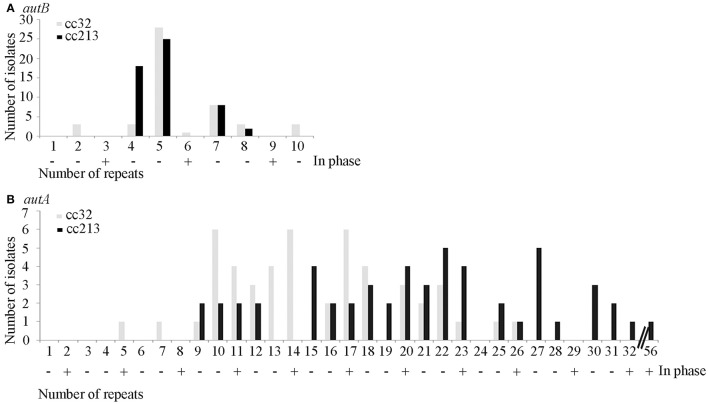
**Variable numbers of AAGC repeats**. The numbers of AAGC repeats were determined in the *autB* **(A)** and *autA* **(B)** genes of 49 cc32 and 53 cc213 meningococcal strains isolated from patients with meningococcal disease. Data for AutA were taken from Arenas et al. (2015). The numbers of repeats that would render the gene in phase are indicated by a plus sign.

The repeat region of the *autB* gene was amplified by PCR and sequenced in a panel of 53 isolates of cc213 and 49 isolates of cc32 collected in The Netherlands between 2000 and 2010 from patients with meningococcal disease. The number of repeat units varied and ranged from 2 to 10 in *autB* and from 5 to 32 in *autA* (Table S3 and Figure 3). These results illustrate that slipped-strand mispairing at the AAGC repeats occurs in both genes and in both clonal complexes. However, in only 1 of the 49 cc32 isolates (2%), i.e., strain 2081107, and none of the 53 cc213 isolates the *autB* gene was in phase. This low frequency contrasts drastically with that observed for *autA*, where the gene was found to be in phase in 28% and 45% for cc213 and cc32 isolates, respectively (Figure 3), i.e., close to the 33% that would be expected in the absence of any selection pressure (Arenas et al., 2015). Thus, the results support the hypothesis that, although slipped-strand mispairing resulting in frameshifts does occur at the AAGC repeats in *autB*, there is a strong selection pressure against the occurrence of a number of repeats that would render the gene in frame in both clonal complexes. Further sequence examination revealed that cc213 isolates do not contain the same additional frameshift that disrupts the *autB* gene downstream of the AAGC repeats in strain M01-240355, suggesting that this feature is not conserved in cc213 strains. Therefore, the gene could potentially be expressed in this cc after slipped-strand mispairing. Interestingly, examination of the sequence after the repeats revealed that isolate 2041085 harbors an AutB2 variant, whilst all other cc213 isolates harbor an AutB1 variant, evidencing horizontal genetic transfer. The full-length *autB* gene was sequenced in three isolates of cc32, i.e., 2081107, which is in frame at the AAGC repeats, and 2061551 and 2070077, which are out of phase. All these isolates revealed only variation in the number of repeat units and no other genetic disruptions or amino-acid substitutions relative to that of strain MC58. Thus, the *autB* gene of isolate 2081107 is intact and, therefore, probably expressed.

### Phase variation of *autB* in carriage isolates

Since the vast majority of isolates analyzed in Tables S2, S3 are from patients with invasive meningococcal disease, our selection of strains may not be representative for those residing in the nasopharynx. Thus, we analyzed the *autB* gene in the genome sequences of a collection of carriage isolates available in a public data base (http://pubmlst.org/software/database/bigsdb/). A total of 207 strains was analyzed. The number of repeat units in the *autB* gene ranged from 2 to 22. In total, 10.6% of these isolates had 3, 6, or 12 repeats that render *autB* in frame. However, in the majority of these strains, the gene is disrupted by downstream-located single-nucleotide insertions and only 2.4% of isolates (28871, 28881, 28942, 28955, 28959) had an undisrupted *autB* gene in frame. Hence, these data do not show obvious differences in possible *autB* expression between carrier and disease isolates.

### Expression of meningococcal AutB

**Figure 4:**
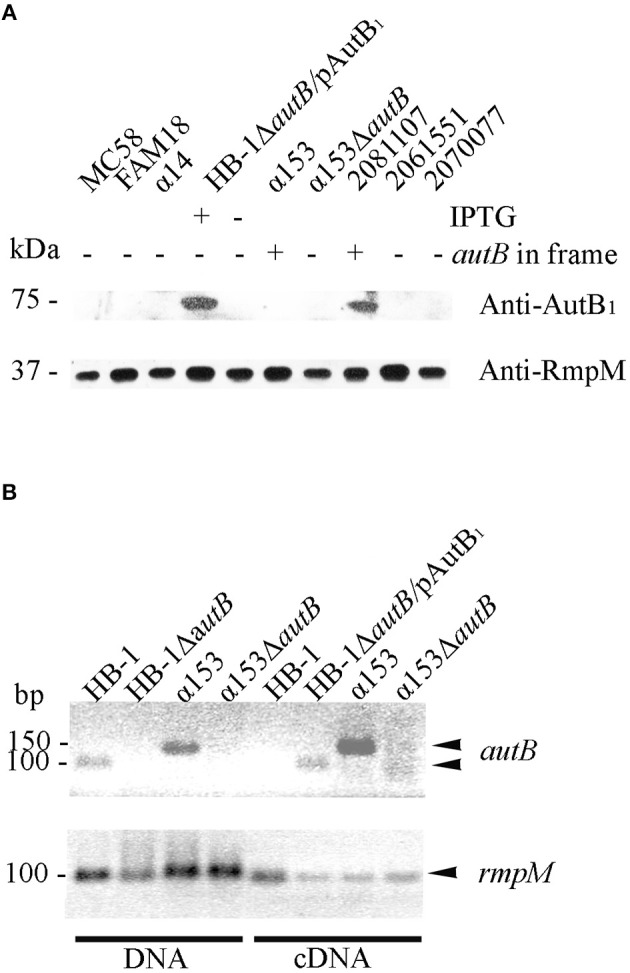
**Expression of AutB. (A)** Western blots of cell envelope preparations of various *N. meningitidis* strains were probed with antisera directed against AutB1 and, as a loading control, against RmpM. HB-1Δ*autB* containing pENAutB_1_ (pAutB_1_) was grown in the presence or absence of IPTG as indicated. Also indicated is whether the chromosomal *autB* gene is in frame or not. Positions of molecular mass standards are shown at the left. Only the relevant parts of the blot are shown. **(B)** Agarose gels showing PCR products obtained with either chromosomal DNA or cDNA as templates as indicated at the bottom. The cDNA was generated from RNA, which was extracted from the *N. meningitidis* strains, using primers for *autB* or *rmpM* as indicated. HB-1Δ*autB* containing pENAutB_1_ was grown in the presence of IPTG. Size standards are shown at the left. Only the relevant parts of the gels are shown.

We next analyzed whether AutB is indeed synthesized in strains where the gene is in phase at the AAGC repeats and has no other genetic disruptions. Plasmids encoding the *autB1* and *autB2* genes from strains 2081107 and α153, respectively, were introduced in HB-1Δ*autB*. Although the *autB* gene is phase off in HB-1 (Table S2), we deleted the chromosomal copy of the gene to avoid genetic exchange with the *autB* gene on the plasmids. Western blot analysis showed a band with an apparent molecular weight of ~73 kDa in strain HB-1Δ*autB* carrying pENAutB_1_ only when the strain was grown in the presence of IPTG (Figure 4A), thus identifying this band AutB_1_. A band of similar size was detected in isolate 2081107, which contains an intact in-frame *autB* gene on the chromosome, but not in preparations of other strains where the *autB1* gene is out of phase (Figure 4A). This result demonstrates that AutB is synthesized in isolate 2081107. Unfortunately, an *autB* mutant of this strain could not be created, because the strain was not transformable. No reaction was observed in strains HB-1Δ*autB* carrying pENAutB_2_ (data not shown) or α153, which is expected to express AutB2 from the chromosome (Figure 4A), presumably due to lack of cross-reactivity of the antiserum, which was raised against a part of AutB1 that shows little sequence similarity to AutB2 (Figure S2). The antiserum did also not react with a preparation of strain α14 (Figure 4A), which expresses AutA, confirming the specificity of the antiserum.

To investigate if *autB2* is expressed in α153, RT-PCR assays were done using primers targeting internal DNA fragments of *autB1* and *autB2*. With chromosomal DNA from HB-1 and α153 as templates, the amplicons for *autB1* and *autB2* were according the expected size, i.e., 100 and 150 bp, respectively (Figure 4B). No amplicons were obtained from RNA preparations as templates (data not shown), but they were obtained from cDNA preparations of strains HB-1Δ*autB* containing pENAutB_1_ and grown in presence of IPTG and α153, but not of strains HB-1 and the α153Δ*autB* mutant (Figure 4B). Primers for *rmpM* yielded an amplicon in all preparations, evidencing that all contained similar amounts of cDNA. The lack of detection of *autB* transcripts in strain HB-1 could be due to degradation of the mRNA by the presence of a premature stop codon resulting from the frameshift at the AAGC repeat region. Together, these data show that *autB* is expressed in at least some meningococcal strains and confirm that its expression is determined by phase variation at the AAGC repeat units.

### Exposure of AutB at the cell surface

**Figure 5:**
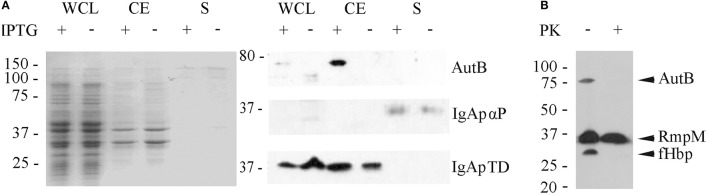
**Localization of AutB. (A)** Strain HB-1Δ*autB* containing pENAutB1 was grown in the presence or absence of IPTG and whole cell lysates (WCL), cell envelopes (CE) and culture supernatants (S) were prepared and separated by SDS-PAGE. The left panel shows a Coomassie-stained gel, and corresponding Western blots with antisera directed against AutB1, and the α-peptide (αP) and the translocator domain (TD) of IgA protease (IgAp) are shown in the right panel. Only the relevant parts of the blots are shown. **(B)** Protease-accessibility assay. Cells of HB-1Δ*autB* containing pENAutB1 were grown in the presence of 0.1 mM IPTG and treated or not with 2 μg/ml of proteinase K (PK). The degradation of AutB, RmpM, and fHbp was analyzed by Western blotting using specific antibodies.

Our Western blotting analysis detected the full-length AutB in cell envelope preparations (Figure 4A). To determine whether the passenger domain may be released from a proportion of the AutB molecules into the extracellular medium, whole cell lysates, cell envelopes and supernatants of strain HB-1Δ*autB* expressing AutB1 from pENAutB_1_ were analyzed by SDS-PAGE and Western blotting. The expected 73-kDa band was detected in cell-envelope preparations but no band reacting with the antiserum was detected in supernatant preparations (Figure 5A). As a control, the secretion of IgA protease was also analyzed using specific antibodies against its translocator domain and the α-peptide, which is released into the milieu after cleavage by NalP (Roussel-Jazédé et al., 2014). As expected, the translocator domain and the α-peptide were detected in the cell envelope fraction and the culture supernatant, respectively (Figure 5A). Hence, the passenger of AutB is not released into the extracellular medium. To confirm its cell-surface exposition, we used proteinase K accessibility assays in intact bacteria (Figure 5B). The protease degraded AutB and fHbp, a surface-exposed lipoprotein that was analyzed as a positive control (Bos et al., 2014). In contrast, the periplasmically exposed outer membrane-associated protein RmpM remained unaffected, confirming the integrity of the outer membrane. Thus, the passenger of AutB is secreted but remains attached via the translocator domain to the cell surface.

### AutB expression impacts biofilm formation

Previously, we demonstrated that AutA has a role in autoaggregation and biofilm formation (Arenas et al., 2015). To investigate whether AutB also has a role in autoaggregation, settling assays were performed. These assays showed a clear effect of the of AutA production on bacterial autoaggregation (Figure S3A); however, such an effect was not observed when *autB1* was expressed from plasmid in HB-1Δ*autB*, and also no difference between α153 and its *autB* mutant derivative was observed (Figure S3A). Thus, in contrast to AutA, synthesis of AutB does not induce strong autoaggregation.

**Figure 6:**
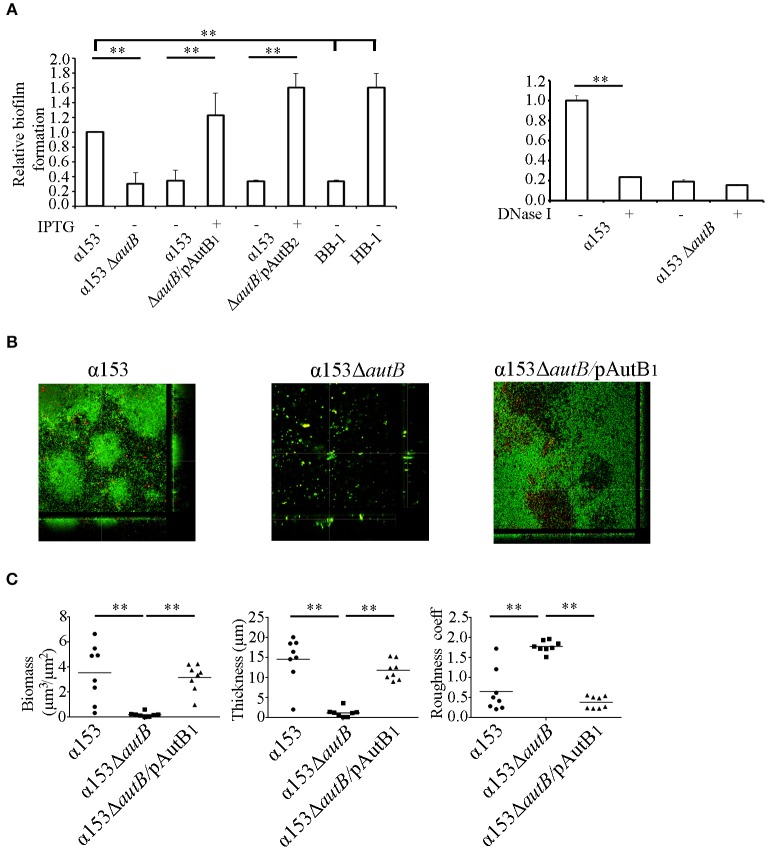
**AutB mediates biofilm formation. (A)** Biofilm formation under static conditions. Bacteria were pre-cultured in TSB with or without 0.1 mM of IPTG and adjusted to an OD_550_ of 1.0. Aliquots were then transferred into 24-well plates and incubated under static conditions for 1 h (left panel). The impact of DNase I on biofilm formation in α153 and its Δ*autB* mutant derivative is shown in the right panel. 100 μg/ml of DNase I was added to the cultures, which were subsequently incubated for 1 h under static conditions. Biofilms formed were quantified after staining with crystal violet by measuring the OD_630_. The data represent means and standard deviations of at least three independent experiments, and values are given as relative to α153, which was set at 1.0. Statistically significant differences between groups are marked with two asterisks (unpaired *t*-test of *P* < 0.01). **(B)** Biofilm formation under flow conditions. Representative pictures are shown of structures of 16 h-old biofilms of α153, α153Δ*autB*, and α153Δ*autB* carrying pENAutB1 cultured in presence of 0.1 mM IPTG (α153Δ*autB*/pAutB_1_). Bacteria were stained with Dead/Live staining for visualization. Green and red bacteria are live and dead cells, respectively. **(C)** Quantification of biomass, thickness, and roughness of 16 h-old biofilms under flow conditions. The biofilm parameters were determined with COMSTAT/MATLAB software. Asterisks indicate statistically significant differences (*P* < 0.0005) between groups calculated by unpaired *t*-test using GraphPad software.

A possible role of AutB in biofilm formation was first studied under static conditions on polystyrene plates. The presence of a capsule is known to inhibit biofilm formation (Yi et al., 2004; Lappann et al., 2006), but α153 is a natural capsule null mutant. We also included the capsule-deficient reference strains HB-1 and BB-1, which were previously used in biofilm assays (Arenas et al., 2013a). After 1 h of incubation, α153 generated a biofilm mass intermediate between that of BB-1 and HB-1 (Figure 6A). Interestingly, deletion of *autB* drastically impaired biofilm formation to a level comparable with that of strain BB-1 (Figure 6A). This phenotype was complemented when either AutB1 or AutB2 was expressed *in trans* from plasmid (Figure 6A), demonstrating that both variants have a similar role.

*N. meningitidis* has two different strategies of biofilm formation, either dependent or independent of eDNA (Lappann et al., 2010). HB-1 and BB-1 follow the eDNA-dependent and -independent routes of biofilm formation, respectively (Arenas et al., 2013a). To investigate whether AutB-mediated biofilm formation is dependent on eDNA, biofilm formation of strain α153 and its *autB* mutant derivative was assessed in the presence of DNase I. This treatment considerably reduced biofilm mass in α153 to the level of the Δ*autB* mutant, but it did not impact on the residual biofilm formation of the mutant (Figure 6A, right panel). This data indicates that AutB-mediated biofilm formation is dependent on eDNA and suggests that the remaining biofilm formation in the *autB* mutant of α153 is eDNA independent. Presumably, AutB at the cell surface binds eDNA that acts as a glue facilitating interbacterial and bacterium-substratum interactions. Previously, the α-peptide of IgA protease and NHBA were reported to represent the main surface-exposed DNA-binding proteins involved in biofilm formation in strain HB-1 (Arenas et al., 2013a). Western blot analysis, however, revealed that the α-peptide of IgA protease is released into the medium in strain α153 (Figure 5A), whilst examination of the α153 genome sequence revealed that the *nhbA* gene is disrupted. Thus, probably, AutB is the main DNA-binding protein in α153 and, therefore, its inactivation has a drastic impact on biofilm formation. To investigate the DNA-binding capacities of AutB, we produced the entire passenger of AutB of strain HB-1 in *E. coli*. The protein formed inclusion bodies, which were purified, but we could not establish proper conditions to fold the protein *in vitro*.

To investigate the influence of AutB synthesis on biofilm formation in more detail, we used a flow-cell model. With this method, the flow in the nasopharynx or the bloodstream is mimicked, and biofilm development can continuously be monitored by microscopy. Biofilms formed by α153 were constituted of round aggregates of different sizes, which were surrounded by single cells (see representative pictures in Figure 6B and the development of the biofilm in Figure S3B). These aggregates fused into larger aggregates, which constituted the biomass of the biofilm (Figure S3B). These biofilm structures had a clearly different appearance than those reported previously for strains HB-1, BB-1, and α14 (Arenas et al., 2013a, 2015). Analysis of the α153Δ*autB* mutant did not reveal structured biofilms; instead, only few single cells or very small aggregates were randomly attached to the substratum (Figure 6B). COMSTAT analysis revealed a biomass and thickness significantly reduced as compared with the wild type (Figure 6C) in accordance with the results of the biofilm assays under static conditions (Figure 6A). Furthermore, biofilm roughness, also determined by COMSTAT, increased significantly. All phenotypic differences were complemented when AutB_1_ was expressed *in trans* from plasmid (Figures 6B,C). These data confirm that AutB is required for biofilm formation and architecture and demonstrate its biological relevance.

### AutB synthesis interferes with meningococcal translocation across epithelial cell layers

**Figure 7:**
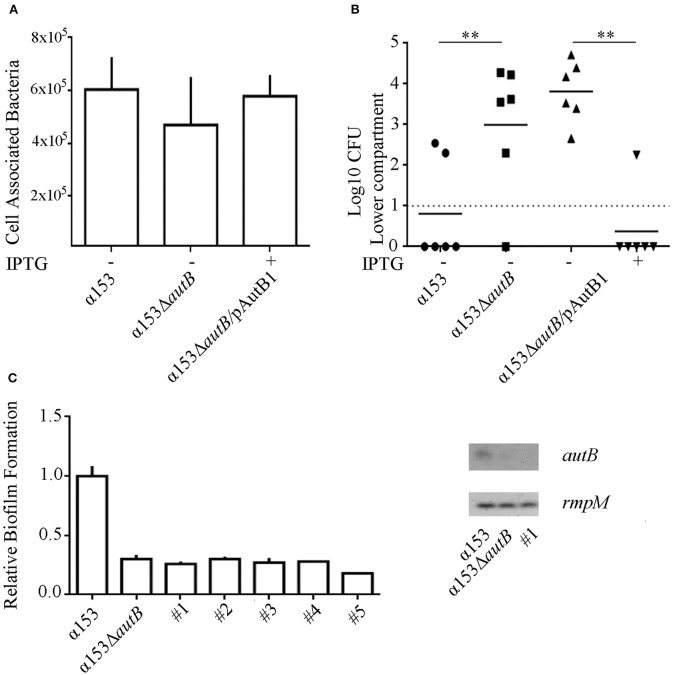
**Inactivation of ***autB*** affects passage of NCI-H292 cell layers. (A)** Adherence of *N. meningitidis* strains α153, α153Δ*autB*, and α153Δ*autB* carrying pENAutB_1_ to NCI-H292 cell layers. The complemented strain was grown in the presence of IPTG. The NCI-H292 cells were incubated with the bacteria for 3 h, washed, and cell-associated CFU were determined after lysing the cells with saponin. Means and standard deviations of three independent experiments performed in duplicate are shown. Differences observed were not statistically significant. **(B)** Passage of cells of strain α153, its Δ*autB* mutant derivative and the complemented mutant through NCI-H292 cell layers. The complemented mutant was grown with or without IPTG as indicated. The passage of bacteria through the cell layers was determined by counting CFUs from the basal compartment 10 h after inoculation. Results of three independent experiments performed in duplicate are shown. Asterisks show statistically significant differences between groups (*P* < 0.001, unpaired *t*-test). Before statistical analysis, CFUs were log10 converted. **(C)** Analysis of clones of cells of strain α153 that reached the trans-epithelial compartment. Several clones were recovered in independent infection experiments (#1–5). Their biofilm-forming capacity was determined as described for Figure 6A and is shown in the graft at the left. Expression of *autB* was determined by RT-PCR as described for Figure 4B, and agarose gels with PCR products obtained with cDNA as templates are shown in the right panel.

To investigate whether the synthesis of AutB also affects the interaction of the bacteria with eukaryotic cells, we first determined the adherence of α153 and its *autB* mutant derivative to NCI-H292 cells by counting the number of cell-associated CFU 3 h after initiating infection. Although adherence of the mutant appeared slightly reduced (~25%), a defect that was complemented when AutB was expressed *in trans* (Figure 7A), this difference was not statistically significant.

Next, we studied the ability of the strains to cross epithelial cell layers, a process necessary for host invasion. NCI-H292 cells were grown for 6 days on a permeable support to generate cellular junctions, as verified by a trans-epithelial resistance of 30 ± 2 Ω × cm^2^, in accordance with values previously reported (van Schilfgaarde et al., 1995). The cell layers were then infected with bacteria and the numbers of bacteria that reached the basal compartment after passage of the cell layers were determined between 6 and 12 h post infection by counting CFU. Cells of the *autB* mutant reached the basal compartment faster than those of the parental strain as demonstrated, for example, by the significantly higher numbers of *autB* mutant bacteria than of wild-type bacteria in the basal compartment 10 h after infection (Figure 7B), a phenotype that was complemented by expression of a functional copy of *autB* from pENAutB_1_. Clones of cells derived from the wild type that reached the trans-epithelial compartment early after infection were collected from different experiments and further analyzed. To investigate whether AutB synthesis and, consequently, their biofilm-formation capacity was altered, we tested biofilm formation under static conditions. Interestingly, all clones had a reduced biofilm-forming capacity like the *autB* mutant (see Figure 7C for representatives). Sequencing of the AAGC region in all clones did not show alterations, suggesting that phase variation was not the origin of such defect. To test whether *autB* expression was lost we analyzed one clone by RT-PCR assays and found no detectable *autB* expression (Figure 7C). Sequencing of the complete *autB* gene and flanking regions, however, did not reveal an obvious reason for this down-regulation, suggesting these clones had acquired a mutation in a regulatory gene. Together, these data demonstrate that *autB* expression negatively affects bacterial epithelial transmigration and suggests that biofilm formation is inversely correlated with invasion.

## Discussion

The *autB* gene is present in bacteria colonizing the human mucosa and is restricted to certain species of the genera *Neisseria* and *Haemophilus*. This distribution was earlier explained by the transfer of the gene from an unidentified microorganism to *H. influenzae* and then to a recent ancestor of both *N. meningitidis* and *N. gonorrhoeae* (Davis et al., 2001). This hypothesis was mainly based on the observation that the gene is placed in *H. influenzae* between the *tmk* and *holB* genes, two genes that are contiguous in many gram-negative bacteria, whilst it is placed in a different context in *N. meningitidis* and *N. gonorrhoeae* but flanked by fragments of the *tmk* and *holB* genes from *H. influenzae* (Figure 1). These observations were made by the examination of the few genome sequences of *Neisseria* and *Haemophilus* strains that were available at that time (Davis et al., 2001). Now, many more genome sequences of both genera are available, and this allowed us to extend the earlier analysis. Indeed, our analysis confirms initial observations and adds new evidence about the evolution and expression of the gene.

First, the *autB* gene is not present in all *H. influenzae* isolates, indicating that the gene does not originate from this species. We found the gene in three other *Haemophilus* spp., i.e., *H. haemolytica, H. parainfluenzae*, and *H. aegyptius*, which are closely related to *H. influenzae*. Also in these species, the gene is found only in a limited number of isolates, indicating that they did not evolve from *autB*-containing *H. influenzae* strains but also acquired the gene by horizontal gene transfer. Second, the gene was ubiquitously present in *N. meningitidis* and *N. gonorrhoeae* genomes, but not in other *Neisseria* spp., consistent with the proposed acquisition of the gene by a recent ancestor of both pathogenic *Neisseria* spp. In agreement with its acquisition by horizontal gene transfer, the GC content of *autB* is substantially lower than that of *Neisseria* genomes. Third, our phylogenetic analysis revealed the existence of three main allelic variants of *autB*. Each variant was not restricted to one single species, but *N. meningitidis, H. influenzae, H. haemolyticus*, and *H. aegyptius* each contained several of these variants, a clear indication that they share a common pool of *autB* genes. In contrast, only *autB1* was detected in *N. gonorrhoeae*, indicating that this species does not participate in the exchange of *autB* variants which is consistent with its different niche. In the evolutionary context, it is also interesting to consider the origin of AutA, which is structurally related to AutB. In contrast to *autB, autA* is present in both pathogenic and commensal *Neisseria* species (Arenas et al., 2015). Therefore, it did not arise by gene duplication after introduction of *autB* into a common ancestor of the pathogenic *Neisseria* spp. Because it is ubiquitous among *Neisseria* spp., it may originate from a common ancestor of *Neisseria* spp. Its subsequent transfer to and evolution in other microorganisms may eventually have resulted in its return as *autB* in *Neisseria*. In agreement with the neisserial origin of *autA* is the GC content of the segments encoding the linker and translocator domains of the protein, which, in contrast to the corresponding domains of *autB*, are in accordance with those of neisserial genomes. However, the GC content of the segment encoding the N-terminal part of the AutA passenger is substantially lower indicating a different evolutionary origin of this part of the gene. Shuffling of domains is also suggested from the phylogenetic analysis of the *autB* genes. The translocator domain of AutB is well conserved, whilst the linker is more conserved than the N-terminal part of the passenger. The N-terminal domain of AutB3 is only distantly related to the corresponding domains of AutB1 and AutB2. In contrast, its linker domain is closely related to that of AutB1, from which it probably evolved after having been linked to a different N-terminal domain.

In all *N. gonorrhoeae* strains examined, the *autB* gene cannot be expressed because of genetic disruptions downstream of the AAGC repeat region, indicating that AutB synthesis is not relevant in the particular niche of this species. In contrast, although phase off in most cases, an intact *autB* gene is preserved in the vast majority of *N. meningitidis* isolates, indicating that it is advantageous for this species to be able to synthesize AutB at some stage in the colonization process. However, it is also evident that it is important for the bacteria to be able to shut off the synthesis of AutB. With only very few exceptions, the number of AAGC repeats renders the gene phase off in meningococci with an intact *autB* gene (Tables S2, S3, Figure 3). Such an extreme preference for the off-phase of a phase-variable gene has, to the best of our knowledge, not been reported before. As compared with *Haemophilus autB*, the number of AAGC repeats in *N. meningitidis autB* is generally lower. A lower number of repeats is expected to result in a reduced frequency of phase variation. However, the variable number of repeats observed in the *autB* gene of isolates of the same clonal complex demonstrates that loss and gain of these repeats through slipped-strand mispairing is still occurring in the meningococcus. When the number of these repeats is reduced to three, the gene is in the on phase and the frequency of phase variation is expected to be very low. In this case, the majority of the isolates have lost the possibility to express the *autB* gene through mutations further downstream in the gene, which accentuates the importance for the bacteria to switch off the expression of the gene.

Why is an intact *autB* gene retained in *N. meningitidis*, while its expression is shut off in the vast majority of the isolates? Like AutB, many other surface-exposed proteins of *N. meningitidis* show phase-variable expression (Saunders et al., 2000), most likely to escape from the immune system. As these proteins have important functions for colonization and persistence in the host, the functions of some of these proteins may be taken over by others when their expression is turned off. The *autB* gene has been considered to be a pseudogene because its expression could not be demonstrated in a limited set of meningococcal strains (Ait-Tahar et al., 2000). We demonstrate here that AutB is synthesized in at least some isolates and that it has a role in biofilm formation presumably by binding eDNA. This role would be equivalent to those of NHBA and the α-peptide of IgA protease (Arenas et al., 2013a). Although the expression of the genes for NHBA and IgA protease has not been reported to be phase variable, these proteins are cleaved from the cell surface by the AT protease NalP (Serruto et al., 2003; van Ulsen et al., 2003; Roussel-Jazédé et al., 2014), the expression of which is phase variable (Saunders et al., 2000). Thus, AutB may serve as a backup mechanism that enables biofilm formation if the amount of cell-surface-exposed NHBA and α-peptide is low.

Then, why is *autB* expression switched off in most isolates? Phase variation by slipped-strand mispairing is a random process and, with the AAGC repeats being located within the coding region, one would expect that 33% of the isolates are in phase on. Such a frequency was indeed found for the *autA* gene (Arenas et al., 2015), but *autB* was found to be in phase on in only ~1% of the same set of isolates, indicating the existence of a strong selection pressure against expression of the gene. Possibly, AutB elicits a strong immune response. Consistent with this hypothesis is the high sequence variability we observed in the passenger domain of AutB, which may reflect antigenic variability due to immune pressure. However, another explanation for selection against *autB* expression in disease isolates is that biofilm formation, whilst presumably important in the colonization of the mucosal surfaces, may be hindering the passage of the epithelial layers to cause invasive disease. Indeed, we observed that AutB synthesis delays the passage of epithelial cell layers *in vitro*. It is noteworthy in this respect that no evidence for selection pressure against *autB* expression was observed in the limited number of *Haemophilus* genomes analyzed. The *H. influenzae* strains in which *autB* (*lav*) was found are nontypeable strains, which seldom cause invasive disease (Davis et al., 2001). Furthermore, *H. haemolyticus* is generally considered as a commensal in the nasopharynx, although also few cases of invasive disease by this bacterium have been reported (Jordan et al., 2011). Thus, these observations suggest a role for AutB during colonization of the nasopharynx and a negative effect of AutB synthesis on bacterial invasion. However, also the vast majority of strains isolated from carriers did not express *autB*. A possible explanation is that the isolation methods for carriage isolates selectively fail to collect AutB-expressing strains. Carriage strains are often isolated from healthy people using swabs. This method underestimates the number of carriers as was demonstrated by Sim et al. (2000), who reported that swabs allowed for the isolation of meningococci from 10% of the people examined whilst meningococci were detected in 45% of these people by immunohistochemistry. In addition, microcolonies were found with the latter technique below the epithelial surface, which could explain the observed underestimation of meningococcal carriage through swab isolation. Thus, it is possible that AutB facilitates microcolony formation in this specific niche.

In summary, we showed here for the first time the synthesis of AutB in some meningococcal isolates and the existence of strong selection pressure against *autB* expression. We also demonstrated the localization of AutB at the bacterial cell surface, its function in biofilm formation, and the negative consequences of its expression on the transit of the bacteria through epithelial cell layers. This work completes the initial characterization of the eight ATs identified so far in available meningococcal genome sequences and provides new insights in the commensal/virulence relationship of pathogenic *Neisseria* and *Haemophilus* species.

## Author contributions

JA, FP, JP, and JT conceived and designed the experiments; JA, SC, AE, and JT performed bioinformatics analysis; JA, FP, PR, and SC performed the experiments; JA, JT analyzed the data and wrote the paper. All authors have read and approved the manuscript.

## Funding

This work was partially supported by Utrecht University. PR, SC were supported by personal fellowships for student mobility (Spanish government) and by an Erasmus fellowship, respectively.

### Conflict of interest statement

The authors declare that the research was conducted in the absence of any commercial or financial relationships that could be construed as a potential conflict of interest.
