# Supplementary material for: Expression of the Gene for Autotransporter AutB of Neisseria meningitidis Affects Biofilm Formation and Epithelial Transmigration
Source: Front Cell Infect Microbiol. 2016 Nov 22;6:162. doi: 10.3389/fcimb.2016.00162 (PMC5118866; doi:10.3389/fcimb.2016.00162)
Supplement: Supplementary file 1 [file Table1.DOCX]

**Table S1.** **Primers used in this study.** Restriction sites used for cloning are indicated and underlined.

| **Goal** | **Primer** | **Sequence** | **Remarks** |
| --- | --- | --- | --- |
| **1.Cloning for expression of AutB_1_ fragment** | NMB01525 FW P1-NdeI | CATATGGTCGGGAATTATACAGAATGGGCTA |  |
|  | NMB1525 R P1-BamHI | GGATCCTTATTCGAAGATGATGTCGGAAGTG |  |
| **2. autB sequencing** |  |  | For cc32 isolates  For cc32 and cc213 isolates |
|  | NMB1525R cc32 only | GTTCCGCCAATAACGCATTG |  |
|  | PautBHB-1R only cc32 | TTCGGTATATTCTTGATCAT |  |
|  |  |  |  |
|  |  |  |  |
|  | PautBHB-1FW and cc213 | CAATGCGTTATTGGCGGAAC |  |
|  | NMB1525FW cc32 and cc213 | TACTGCATTAATTTTTGAGA |  |
|  |  |  |  |
|  | NMB1524FW both | GCGCGCCACGCGAGAAACAGCGGTTGA |  |
|  | PautBpass cc32 and cc213 | CCAAGCTCACTAATGATGAA |  |
|  | PautBb-barrel R cc32 both | GCTTGAGCCTGTTGTGCATA |  |
|  |  |  |  |
|  |  |  |  |
| **3. Constructing *autB* knockout mutant** | NMB1525FWF1-BglII | GCGCGCAGATCTTGGAAACAGTGCTTCAGACG | Upstream flanking region |
|  | NMB1525R –NcoI | GCGCGCCCATGGTCCTTTGTCAAGTAAAAATA |  |
|  | NMB1525 FW F2 - NdeI | GCGCGCCATATGGTAACAAAATAAAATCGGCA | Downstream flanking region |
|  | NMB1524 RF2–EcoRV | GCGCGCGATATCTCTGGGCGACGGCAGCCTGC |  |
| **4. To check the knockout mutants** | pNMB1524R | GCGCGCACGCGAGAAACAGCGGTTGA |  |
|  | pNMB1526FW | GCGCGCCAAAGGCATACAAATCGTCT |  |
| **5. Constructing AutB expression plasmids** | Fw autB 1.32 NdeI | GCGCGCCATATGAAACTCGAAGCAAGCAAGCAAGCAAGCAAGCAGAAGTTTAAAAAATCATT | To amplify  *autB1* and *autB2* |
|  | RautB 1.32 AatII | GCGCGCGACGTCTTAAAACGTCCACTGTAAAT |  |
| **6. RT-PCR** | RT PCR autB1 FW | GCAAAAAGAAATGGGGACAC | To amplify *autB_1_* |
|  | RT PCR autB1R | GCTTGGCAAGTACTATAATCACG |  |
|  | RT PCR autB2 R | TAACTAACATCAGAATCCCAACGAGGCTCCGC | To amplify *autB*_2_ |
|  | RT PCR autB2 FW | GCTATATATGTAGATTGGTCTATATTCTCTCT |  |
|  | RT-RmpMFW | GCAACTTCGGCTTCACAAAC | To amplify *rmpM* |
|  | RT-RmpMR | CGCCAGGGTTTTCCAGTCACGA |  |
