# Supplementary material for: Expression of the Gene for Autotransporter AutB of Neisseria meningitidis Affects Biofilm Formation and Epithelial Transmigration
Source: Front Cell Infect Microbiol. 2016 Nov 22;6:162. doi: 10.3389/fcimb.2016.00162 (PMC5118866; doi:10.3389/fcimb.2016.00162)
Supplement: Supplementary file 2 [file Table2.DOC]

| **Strain**  **Table S2.** **Characteristics of *autB* in available genome sequences.** Listed are homologous genes obtained in BLASTnt searches using as queries the full-length *autB* sequence of MC58 or its passenger-encoding domain. The gene locus, number of tetranucleotide repeat units at the 5’ end and the resulting in/out phase are given. | **Clonal**  **Complex ^a^** | | | **Gene Locus Localization ^b^** | | **Phase ^c^** | **Rn^d^** | **Remarks^e^** | **Expression^f^** | |  |
| --- | --- | --- | --- | --- | --- | --- | --- | --- | --- | --- | --- |
| ***N. meningitidis*** | | |  |  |  |  |  |  |  | | |
| 2000063 | 5 | | | NM2000063_1495 | 14685-16701 | Out | 4 | B1 | (+) | | |
| 2000080 | 5 | | | NM2000080_1467 | 15433-17449 | Out | 4 | B1 | (+) | | |
| 2001001 | 11 | | | NM2001001_1475 | 15340-17407 | Out | 13 | B1 | (+) | | |
| 2001212 | 5 | | | NM2001212_1347 | 25725-27741 | Out | 4 | B1 | (+) | | |
| 2002004 | 11 | | | NM2002004_1556 | 6994-9037 | Out | 7 | B1 | (+) | | |
| 2002007 | 5 | | | NM2002007_1471 | 15118-17134 | Out | 4 | B1 | (+) | | |
| 2002020 | 32 | | | NM2002020_1526 | 15399-17451 | Out | 10 | B1 | (+) | | |
| 2002030 | 32 | | | NM2002030_1510 | 15243-17287 | Out | 8 | B1 | (+) | | |
| 2002038 | 181 | | | NM2002038_1987 | 148444-150458 | Out | 4 | B1 | (+) | | |
| 2003022 | 5 | | | NM2003022_1474 | 15481-17497 | Out | 4 | B1 | (+) | | |
| 2003051 | NA | | | NM2003051_1464 | 29860-31915 | Out | 10 | B1 | (+) | | |
| 2004032 | NA | | | NM2004032_1454 | 19623-21651 | In | 3 | Fs, B1 | - | | |
| 2004085 | 5 | | | NM2004085_1487 | 15418-17434 | Out | 4 | B1 | (+) | | |
| 2004090 | 5 | | | NM2004090_1547 | 56432-58448 | Out | 4 | B1 | (+) | | |
| 2005040 | 11 | | | NM2005040_1460 | 26442-28487 | Out | 7 | B1 | (+) | | |
| 2005172 | 181 | | | NM2005172_1539 | 15181-17198 | Out | 5 | B1 | (+) | | |
| 2006087 | 181 | | | NM2006087_1583 | 60141-62154 | Out | 4 | B1 | (+) | | |
| 2007056 | 5 | | | NM2007056_1656 | 26636-28652 | Out | 4 | B1 | (+) | | |
| 2007461 | NA | | | NM2007461_1447 | 15331-17359 | In | 3 | Fs, B1 | - | | |
| 2008223 | 181 | | | NM2008223_1488 | 15163-17180 | Out | 5 | B1 | (+) | | |
| 510612 | NA | | | NMA510612_1942 | 1668511-1670527 | Out | 4 | B1 | (+) | | |
| 63006 | 4 | | | NM63006_1429 | 56859-58875 | Out | 4 | B1 | (+) | | |
| 63049 | 4 | | | NM63049_1430 | 58164-60180 | Out | 4 | B1 | (+) | | |
| 64182 | 4 | | | NM64182_1410 | 15516-17532 | Out | 4 | B1 | (+) | | |
| 65012 | 4 | | | NM65012_1463 | 27693-29709 | Out | 4 | B1 | (+) | | |
| 65014 | 4 | | | NM65014_0472 | 56471-58487 | Out | 4 | B1 | (+) | | |
| 69166 | 1 | | | NMEN69166_1554 | 132569-134598 | Out | 5 | B1 | (+) | | |
| 75643 | 5 | | | NM75643_1423 | 9625-11641 | Out | 4 | B1 | (+) | | |
| 75689 | 5 | | | NM75689_1525 | 15366-17382 | Out | 4 | B1 | (+) | | |
| 77221 | NA | | | NM77221_1843 | 58685-60716  854588-856760 | Out | 4 | B1 | (+) | | |
| 8013 | 18 | | | NMV_0866 |  | In | 3 | Fs, B2 | - | | |
| 80179 | 178 | | | NMEN80179_0948 | 222995-225182 | Out | 7 | B2 | (+) | | |
| 88050 | 5 | | | NM88050_1428 | 131280-133296 | Out | 4 | B1 | (+) | | |
| 92045 | NA | | | NMEN92045_1521 | 127884-130071 | Out | 7 | B2 | (+) | | |
| 93003 | NA | | | NMEN93003_1462 | 130019-132050 | Out | 4 | B1 | (+) | | |
| 93004 | NA | | | NMEN93004_1640 | 14997-17052 | Out | 10 | B1 | (+) | | |
| 94018 | NA | | | NM94018_1442 | 15055-17071 | Out | 4 | B1 | (+) | | |
| 9506 | 32 | | | NM9506_1430 | 130054-132094 | Out | 7 | B1 | (+) | | |
| 96023 | 5 | | | NM96023_1283 | 55679-57695 | Out | 4 | B1 | (+) | | |
| 96024 | 5 | | | NM96024_1473 | 15245-17261 | Out | 4 | B1 | (+) | | |
| 96037 | 41/44 | | | NM96037_1550 | 15484-17516 | Out | 5 | B1 | (+) | | |
| 961-5945 | 8 | | | NMB9615945_0721 | 1895-4121 | Out | 4 | B2 | (+) | | |
| 97008 | 5 | | | NM97008_1505 | 15205-17221 | Out | 4 | B1 | (+) | | |
| 97014 | 181 | | | NM97014_1669 | 106224-108241 | Out | 5 | B1 | (+) | | |
| 97018 | 181 | | | NM97018_1500 | 15111-17127 | Out | 4 | B1 | (+) | | |
| 97020 | 5 | | | NM97020_1515 | 104184-106200 | Out | 4 | B1 | (+) | | |
| 97021 | 181 | | | NM97021_1491 | 61222-63239 | Out | 5 | B1 | (+) | | |
| 97027 | 4 | | | NM97027_1510 | 20385-22401 | Out | 4 | B1 | (+) | | |
| 9757 | 32 | | | NM9757_1466 | 60318-62358 | Out | 7 | B1 | (+) | | |
| 98002 | 181 | | | NM98002_1519 | 15386-17403 | Out | 5 | B1 | (+) | | |
| 98005 | 5 | | | NM98005_1447 | 15359-17375 | Out | 4 | B1 | (+) | | |
| Alpha14 | 53 | | | NMO_1346 | 1427984-1430019 | Out | 5 | B1 | (+) | | |
| Alpha153 | 60 | | | NME_1269 | 1120804-1122993 | In | 3 | B2 | + | | |
| Alpha704 | NA | | | BN21_1109 | 14903-16976 | Out | 19 | B1 | (+) | | |
| Alpha710 | 41/44 | | | NMBB_1754A | 1555869-1557892 | In | 3 | Fs, B1 | - | | |
| ATCC 13091 | NA | | | HMPREF0602_0555 | 16252-18287 | Out | 8 | B1 | (+) | | |
| CU385 | 32 | | | NMBCU385_0667 | 28798-30838 | Out | 7 | B1 | (+) | | |
| ES14902 | 11 | | | NMBES14902_0756 | 17004-19039 | Out | 5 | B1 | (+) | | |
| FAM18 | 11 | | | NMC1454 | 1461459-1463503 | Out | 7 | PSC, B1 | - | | |
| G2136 | 8 | | | NMBG2136_1406 | 1477016-1479051 | Out | 5 | B1 | (+) | | |
| H44/76 | 32 | | | NMBH4476_0704 | 755048-757080 | Out | 5 | B1 | (+) | | |
| L91543 | NA | | | QP84_04950 | 58275-60322 | Out | 8 | B1 | (+) | | |
| LNP21362 | 32 | | | N875_10315 | 1999386-2001418 | Out | 5 | B1 | (+) | | |
| LNP27256 | 11 | | | N872_02230 | 33460-35487 | In | 3 | B1 | + | | |
| M01-240013 | 269 | | | NMBM01240013_0759 | 4528-6536 | Out | 5 | B1 | (+) | | |
| M01-240149 | 41/44 | | | NMBM01240149_0644 | 736223-738258 | Out | 5 | B1 | (+) | | |
| M01-240355 | 213 | | | NMBM01240355_1448 | 1561622-1563644 | In | 3 | Fs, B1 | - | | |
| M04-240196 | 269 | | | NMBM04240196_0694 | 744515-747390 | In | 3 | t, B1 | - | | |
| NM045 | 41/44 | | | NM045_1542 | 15444-17476 | Out | 5 | B1 | (+) | | |
| NM0552 | 41/44 | | | NM0552_1523 | 15340-17371 | Out | 4 | B1 | (+) | | |
| M0579 | 41/44 | | | NMBM0579_0699 | 23741-26010 | Out | 5 | B2 | (+) | | |
| M10208 | 11 | | | LD07_00845 | 157187-159230 | Out | 7 | B1 | (+) | | |
| M13255 | 32 | | | NMM13255_0681 | 120233-122273 | Out | 7 | B1 | (+) | | |
| M13265 | 32 | | | NMM13265_1520 | 15431-17471 | Out | 7 | B1 | (+) | | |
| M13399 | 269 | | | NMBM13399_0704 | 64201-66205 | Out | 4 | B1 | (+) | | |
| M6190 | 11 | | | NMBM6190_0594 | 2124-4159 | Out | 5 | B1 | (+) | | |
| MC58 | 32 | | | NMB1525 | 1574268-1576300 | Out | 5 | B1 | (+) | | |
| N1568 | 181 | | | NMXN1568_0647 | 23551-25568 | Out | 5 | B1 | (+) | | |
| NM003 | 41/44 | | | NM003_1481 | 15811-17843 | Out | 5 | B1 | (+) | | |
| NM140 | 103 | | | NMEN140_1401 | 26648-28920 | Out | 8 | B2 | (+) | | |
| NM1476 | 32 | | | NM1476_1502 | 22840-24892 | Out | 10 | B1 | (+) | | |
| NM151 | 175 | | | NM151_1552 | 14901-16936 | Out | 5 | B1 | (+) | | |
| NM165 | 23 | | | NM165_1400 | 15327-17355 | In | 3 | Fs, B2 | - | | |
| NM183 | 32 | | | NMEN183_1430 | 60802-63070 | Out | 7 | B1 | (+) | | |
| NM2657 | 60 | | | NMEN2657_1417 | 26753-28946 | Out | 4 | B2 | (+) | | |
| NM2781 | 103 | | | NMEN2781_1629 | 60318-62586 | Out | 7 | B2 | (+) | | |
| NM2795 | 198 | | | NMEN2795_1543 | 100323-102333 | Out | 2 | Fs, B1 | - | | |
| NM2866 | NA | | | NM2866_1550 | 15447-17519 | Out | 4 | Fs, B1 | - | | |
| NM3001 | 1157 | | | NMEN3001_0443 | 194587-196613 | Out | 2 | B1 | (+) | | |
| NM3042 | 23 | | | NM3042_1483 | 26102-28130 | In | 3 | Fs, B1 | - | | |
| NM3081 | NA | | | NMEN3081_1649 | 32552-34565 | Out | 4 | B1 | (+) | | |
| NM3139 | 41/44 | | | NM3139_1495 | 15097-17137 | Out | 7 | B1 | (+) | | |
| NM3141 | 32 | | | NM3141_1545 | 15413-17453 | Out | 7 | B1 | (+) | | |
| NM3173 | 32 | | | NM3173_1482 | 15072-17112 | Out | 7 | B1 | (+) | | |
| NM3223 | 23 | | | NM3223_1391 | 3016-5044 | In | 3 | Fs, B1 | - | | |
| NM3230 | 41/44 | | | NM3230_1537 | 6727-8770 | Out | 8 | B1 | (+) | | |
| NM35 | 11 | | | NM35_1472 | 6609-8656 | Out | 8 | B1 | (+) | | |
| NM36 | 11 | | | NM36_1461 | 15563-17610 | Out | 8 | B1 | (+) | | |
| NM3642 | 5 | | | NMNM3642_1511 | 102223-104239 | Out | 4 | B1 | (+) | | |
| NM3652 | 5 | | | NMNM3652_1392 | 25888-27904 | Out | 4 | B1 | (+) | | |
| NM3681 | 11 | | | LA54_09770 | 374868-376915 | Out | 8 | B1 | (+) | | |
| NM3682 | 11 | | | LA58_08590 | 1551465-1553508 | Out | 7 | B1 | (+) | | |
| NM3683 | 11 | | | LC14_07955 | 1421704-1423747 | Out | 7 | B1 | (+) | | |
| NM3686 | 11 | | | LA50_10185 | 1828806-1830865 | Out | 11 | B1 | (+) | | |
| NM3687 | 11 | | | LA53_08975 | 1465406-1467449 | Out | 7 | B1 | (+) | | |
| NM418 | 32 | | | NMNM418_1544 | 58448-60488 | Out | 7 | B1 | (+) | | |
| NM422 | 32 | | | NMNM422_1508 | 57577-59617 | Out | 7 | B1 | (+) | | |
| NM477 | 32 | | | NM477_1593 | 14859-16899 | Out | 7 | B1 | (+) | | |
| NM51 | 23 | | | NM51_1444 | 26180-28238 | In | 3 | Fs, B1 | - | | |
| NM518 | 41/44 | | | NM518_1535 | 15535-17570 | Out | 5 | B1 | (+) | | |
| NM604 | 5 | | | NM604_1526 | 24957-26972 | Out | 4 | B1 | (+) | | |
| NM606 | NA | | | NM606_1478 | 18755-20771 | Out | 4 | B1 | (+) | | |
| NM607 | 5 | | | NM607_1511 | 14892-16908 | Out | 4 | B1 | (+) | | |
| NZ-05/33 | 41/44 | | | NMBNZ0533_1501 | 1596963-1599006 | Out | 7 | B1 | (+) | | |
| OX99-30304 | 41/44 | | | NMBOX9930304_0642 | 1481-3521 | Out | 7 | B1 | (+) | | |
| WUE 2594 | 5 | | | NMAA_1219 | 1432114-1434130 | Out | 4 | B1 | (+) | | |
| Z2491 | 4 | | | NMA1725 | 1656349-1658365 | Out | 4 | B1 | (+) | | |
| ***N. gonorrhoeae*** | |  | |  |  |  |  |  |  | | |
| 1291 | NA | | | NGAG_00821 | 21467-23496 | Out | 2 | Fs, B1 | - | | |
| 35/02 | NA | | | NGBG_00918 | 20336-22365 | In | 3 | Fs, B1 | - | | |
| DGI18 | NA | | | NA | 6777-8806 | In | 3 | Fs, B1 | - | | |
| DGI2 | NA | | | NGMG_01402 | 25789-27818 | Out | 2 | Fs, B1 | - | | |
| F62 | NA | | | NA | 17248-19277 | In | 3 | Fs, B1 | - | | |
| FA 1090 | NA | | | NA | 954930-956959 | In | 3 | Fs, B1 | - | | |
| FA19 | NA | | | NGEG_04763/4 | 383468-385494 | Out | 2 | Fs, B1 | - | | |
| FA6140 | NA | | | NA | 28948-30977 | In | 3 | Fs, B1 | - |  | |
| MS11 | NA | | | NA | 289933-291961 | In | 3 | Fs, B1 | - |  | |
| PID1 | NA | | | NGHG_00113 | 21468-23497  17726-19755  6779-8808 | In | 3 | Fs, B1 | - |  | |
| PID18 | NA | | | NGGG_00792 |  | Out | 2 | Fs, B1 | - |  | |
| PID24-1 | NA | | | NA |  | In | 3 | Fs, B1 | - |  | |
| PID332 | NA | | | NGJG_00911 | 21467-23493 | Out | 2 | Fs, B1 | - |  | |
| SK-92-679 | NA | | | NA | 19353-21382 | In | 3 | Fs, B1 | - |  | |
| SK-93-1035 | NA | | | NGLG_00907 | 19361-21390 | Out | 2 | Fs, B1 | - |  | |
| NCCP11945 | NA | | | NGK_0807 | 656985-659014 | In | 3 | Fs, B1 | - |  | |
|  |  | | |  |  |  |  |  |  |  | |
| ***H. influenzae*** | | | |  |  |  |  |  |  |  | |
|  |  | | |  |  |  |  |  |  |  | |
| 86-028NP | NA | | | NTHI0585 | 544254-546377 | In | 30 | B1 | + |  | |
| PittGG | NA | | | CGSHiGG_05515 | 1001211-1003522 | Out | 17 | B2 | (+) |  | |
| R2866 | NA | | | R2866_0118 | 119734-121811 | Out | 20 | B1 | (+) |  | |
| F3031 | NA | | | HIBPF_RS09005 | 1795716-1797837 | Out | 28 | B3 | (+) |  | |
| F3047 | NA | | | HICON_09610 | 751946-754026 | Out | 20 | B3 | (+) |  | |
| Hi375 | NA | | | NF38_02785 | 565736-568051 | In | 30 | B2 | + |  | |
| 477 | NA | | | NTHI477_01570 | 1587419-1589490 | Out | 17 | B1 | (+) |  | |
|  |  | | |  |  |  |  |  |  |  | |
| ***H. haemolyticus*** | | | |  |  |  |  |  |  |  | |
|  |  | | |  |  |  |  |  |  |  | |
| M19501 | NA | | | GG9_1580 | 47955- 50189 | In | 18 | B2 | + |  | |
| 27P25 | NA | | | AAX15_06810 | 19058-21148 | In | 21 | B3 | + |  | |
| ***H. parainfluenzae*** | | | |  |  |  |  |  |  |  | |
|  | | | |  |  |  |  |  |  |  | |
| 614_HPAR^g^  ***H. aegyptius***  ATCC 1116  CCUG 26840  CCUG 628 | NA  NA  NA  NA | | | ADG89_RS00295  HMPREF9095_0139  A9520_01365  A9506_07150 | 80369-82473  82261-84361  233034-235300 | Out  Out  Out | 26  25  5 | B1  B3  B3  B2 | (+)  (+)  (+) |  | |
|  |  | | |  |  |  |  |  |  |  | |

^a^ NA, Not available

^b^ The exact position of the entire gene in the genome sequence is specified.

^c^ In/Out of frame was considered according to the number of AAGC repeats.

^d^ Rn, number of tetranucleotide repeats.

^e^ Other genetic features. Sequence variations are mostly located in the 5´ region, encoding the predicted signal sequence and passenger domain. The passenger domains of AutB proteins are clustered in three variants indicated as B1, B2 and B3; a representative cluster analysis is shown in Fig 2. The presence of frameshifts (Fs), transposase elements (t) and premature stop codons (PSC) that cause gene disruption is indicated.

^f^ +, a full-length AutB is expected to be synthesized; (+), a full-length AutB could be synthesized after phase variation in the repeat region; -, no full-length AutB can be synthesized even if the gene is in phase at the tetranucleotide repeats because of the presence of other gene-disruptive features.

^g^ Incomplete genome sequence
