# Supplementary material for: Expression of the Gene for Autotransporter AutB of Neisseria meningitidis Affects Biofilm Formation and Epithelial Transmigration
Source: Front Cell Infect Microbiol. 2016 Nov 22;6:162. doi: 10.3389/fcimb.2016.00162 (PMC5118866; doi:10.3389/fcimb.2016.00162)
Supplement: Supplementary file 3 [file Table3.DOCX]

Table S3. Number of AAGC repeats in the *autA* and *autB* genes in cc32 and cc213 clinical isolates of *N. meningitidis.*

| ***autA autB*** | | | | | | | | | | |
| --- | --- | --- | --- | --- | --- | --- | --- | --- | --- | --- |
| **Isolate** | **Year** | **cc** | **ST^a^** | **FetA^b^** | **VR1^b^** | **VR2^b^** | **#Rn^c^** | **Phase^d^** | **#Rn^c^** | **Phase^d^** |
| 2104366 | 2010 | 32 | 34 | F5-1 | 5-2 | 10 | 10 | Out | 5 | Out |
| 2093472 | 2009 | 32 | 34 | F5-1 | 5-2 | 10 | 18 | Out | 5 | Out |
| 2090516 | 2009 | 32 | 32 | F3-3 | 7 | 16 | 9 | Out | 5 | Out |
| 2090354 | 2009 | 32 | 3720 | F5-1 | 5-2 | 10 | 17 | In | 7 | Out |
| 2090249 | 2009 | 32 | 34 | F5-1 | 5-2 | 10 | 17 | In | 8 | Out |
| 2090110 | 2009 | 32 | 33 | F5-1 | 19-1 | 15 | 23 | In | 5 | Out |
| 2082130 | 2008 | 32 | 34 | F5-1 | 5-2 | 10 | 13 | Out | 5 | Out |
| 2081656 | 2008 | 32 | 32 | F3-3 | 7 | 16-32 | 11 | In | 5 | Out |
| 2081186 | 2008 | 32 | 34 | F5-1 | 5-2 | 10 | 22 | Out | 5 | Out |
| 2081107 | 2008 | 32 | 34 | F5-1 | 19 | 15 | 22 | Out | 6 | In |
| 2080724 | 2008 | 32 | 7884 | F3-3 | 7 | 16-97 | 11 | In | 5 | Out |
| 2080723 | 2008 | 32 | 32 | F3-3 | 7 | 16-97 | 12 | Out | 5 | Out |
| 2080672 | 2008 | 32 | 32 | F3-3 | 7-2 | 16 | 17 | In | 2 | Out |
| 2080654 | 2008 | 32 | 34 | F5-1 | 5-2 | 10 | 20 | In | 5 | Out |
| 2080151 | 2008 | 32 | 32 | F3-3 | 21 | 16 | 21 | Out | 5 | Out |
| 2080072 | 2008 | 32 | 6871 | F1-5 | 7 | 16-2 | 17 | In | 10 | Out |
| 2080070 | 2008 | 32 | 32 | F3-3 | 7 | 16-38 | 11 | In | 7 | Out |
| 2072036 | 2007 | 32 | 34 | F5-1 | 5-2 | 10 | 14 | In | 5 | Out |
| 2071428 | 2007 | 32 | 32 | F3-3 | 7-2 | 16-43 | 13 | Out | 2 | Out |
| 2071291 | 2007 | 32 | 3720 | F5-1 | 22 | 9 | 14 | In | 10 | Out |
| 2071283 | 2007 | 32 | 3720 | F5-1 | 5-2 | 10 | 20 | In | 8 | Out |
| 2071282 | 2007 | 32 | 259 | F3-3 | 7 | 16 | 10 | Out | 7 | Out |
| 2070947 | 2007 | 32 | 6299 | F3-3 | 7 | 16-32 | 13 | Out | 7 | Out |
| 2070946 | 2007 | 32 | 6299 | F3-3 | 7 | 16-32 | 14 | In | 7 | Out |
| 2070929 | 2007 | 32 | 32 | F3-3 | 7 | 16 | 26 | In | 4 | Out |
| 2070582 | 2007 | 32 | 33 | F5-1 | 19 | 15 | 17 | In | 7 | Out |
| 2070568 | 2007 | 32 | 32 | F3-3 | 7-2 | 16-32 | 14 | In | 5 | Out |
| 2070472 | 2007 | 32 | 6292 | F5-1 | 5 | 2 | 5 | In | 5 | Out |
| 2070077 | 2007 | 32 | 34 | F5-1 | 5-2 | 10 | 14 | In | 5 | Out |
| 2061646 | 2006 | 32 | 32 | F3-3 | 7 | 16-32 | 10 | Out | 5 | Out |
| 2061468 | 2006 | 32 | 34 | F5-1 | 5-2 | 10 | 10 | Out | 5 | Out |
| 2061322 | 2006 | 32 | 32 | F3-3 | 7 | 16-85 | 12 | Out | 5 | Out |
| 2061227 | 2006 | 32 | 5955 | NA | 19 | 15 | 7 | Out | 10 | Out |
| 2021127 | 2002 | 32 | NA | NA | NA | NA | 20 | In | 5 | Out |
| 2000606 | 2000 | 32 | NA | NA | NA | NA | 12 | Out | 5 | Out |
| 2061546 | 2006 | 32 | 34 | F5-1 | 5-2 | 10 | 16 | Out | 5 | Out |
| 2091198 | 2009 | 32 | 3720 | F5-1 | 5-2 | 10 | 10 | Out | 4 | Out |
| 2090092 | 2009 | 32 | 34 | F5-1 | 5-2 | 10 | 25 | Out | 5 | Out |
| 2081105 | 2008 | 32 | 32 | F3-3 | 7-2 | 16-99 | 18 | Out | 2 | Out |
| 2080564 | 2008 | 32 | 34 | F5-1 | 21 | 16 | 17 | In | 5 | Out |
| 2070808 | 2007 | 32 | 3720 | F5-1 | 22 | 9 | 16 | Out | 8 | Out |
| 2090919 | 2009 | 32 | 34 | F5-50 | 5-2 | 10 | 10 | Out | 7 | Out |
| 2082361 | 2008 | 32 | 32 | F3-3 | 7 | 16-32 | 11 | In | 5 | Out |
| 2081056 | 2008 | 32 | 34 | F5-1 | 5-2 | 10 | 21 | Out | 5 | Out |
| 2071749 | 2007 | 32 | 34 | F5-1 | 5-2 | 10 | 18 | Out | 7 | Out |
| 2071002 | 2007 | 32 | 34 | F5-50 | 5-2 | 10 | 22 | Out | 5 | Out |
| 2070133 | 2007 | 32 | 32 | F3-3 | 7 | 16 | 18 | Out | 4 | Out |
| 2061551 | 2006 | 32 | 32 | F3-3 | 7 | 16-32 | 14 | In | 5 | Out |
| 2070755 | 2007 | 32 | 4949 | F1-80 | 5-1 | 0-2 | 13 | Out | 5 | Out |
| 2021086 | 2002 | 213 | 213 | F5-5 | 22 | 14 | 27 | Out | 5 | Out |
| 2020383 | 2002 | 213 | 213 | F1-7 | 22 | 14 | 21 | Out | 5 | Out |
| 2012072 | 2001 | 213 | 213 | NA | 22 | 14 | 22 | Out | 4 | Out |
| 2011319 | 2001 | 213 | 213 | NA | 22 | 14 | 9 | Out | 5 | Out |
| 2010748 | 2001 | 213 | 213 | NA | 22 | 14 | 23 | In | 5 | Out |
| 2001778 | 2000 | 213 | 213 | NA | 22 | 14 | 23 | In | 4 | Out |
| 2001606 | 2000 | 213 | 213 | NA | 22 | 14 | 20 | In | 5 | Out |
| 2001199 | 2000 | 213 | 213 | NA | 22 | 14 | 18 | Out | 5 | Out |
| 2031441 | 2003 | 213 | 213 | NA | 22 | 14 | 23 | In | 4 | Out |
| 2030259 | 2003 | 213 | 213 | F5-5 | 22 | 14 | 27 | Out | 7 | Out |
| 2021828 | 2002 | 213 | 213 | NA | 22 | 14 | 56 | In | 7 | Out |
| 2021590 | 2002 | 213 | 213 | F5-5 | 22 | 14 | 23 | In | 4 | Out |
| 2041085 | 2004 | 213 | 213 | F5-5 | 22 | 14 | 15 | Out | 4 | Out |
| 2040789 | 2004 | 213 | 213 | F1-7 | 22 | 14 | 17 | In | 4 | Out |
| 2040964 | 2004 | 213 | 213 | F5-5 | 22 | 14 | 25 | Out | 5 | Out |
| 2040042 | 2004 | 213 | 213 | F5-5 | 22 | 14 | 22 | Out | 5 | Out |
| 2032691 | 2003 | 213 | 213 | F1-30 | 22 | 14 | 10 | Out | 5 | Out |
| 2032080 | 2003 | 213 | 213 | NA | 22 | 14 | 19 | Out | 4 | Out |
| 2032022 | 2003 | 213 | 213 | F5-5 | 22 | 14 | 30 | Out | 4 | Out |
| 2041499 | 2004 | 213 | 213 | F5-17 | 22 | 14 | 18 | Out | 4 | Out |
| 2041443 | 2004 | 213 | 213 | F5-5 | 22 | 14 | 22 | Out | 5 | Out |
| 2041343 | 2004 | 213 | 213 | F5-5 | 22 | 14 | 31 | Out | 5 | Out |
| 2041156 | 2004 | 213 | 213 | F5-5 | 22 | 14 | 30 | Out | 5 | Out |
| 2041096 | 2004 | 213 | 213 | F5-5 | 22 | 14 | 31 | Out | 5 | Out |
| 2050767 | 2005 | 213 | 213 | F5-5 | 22 | 14 | 21 | Out | 5 | Out |
| 2050747 | 2005 | 213 | 213 | F5-5 | 22 | 14 | 22 | Out | 5 | Out |
| 2050693 | 2005 | 213 | 213 | F5-5 | 22 | 14 | 27 | Out | 5 | Out |
| 2050425 | 2005 | 213 | 213 | F5-5 | 22 | 14 | 22 | Out | 5 | Out |
| 2050392 | 2005 | 213 | 213 | F5-5 | 22 | 14-12 | 28 | Out | 5 | Out |
| 2060786 | 2006 | 213 | 213 | F5-5 | 22 | 14 | 16 | Out | 5 | Out |
| 2060456 | 2006 | 213 | 213 | F5-5 | 22 | 14 | 18 | Out | 5 | Out |
| 2060139 | 2006 | 213 | 213 | F5-5 | 22 | 14 | 11 | In | 5 | Out |
| 2051278 | 2005 | 213 | 213 | F5-5 | 22 | 14 | 15 | Out | 4 | Out |
| 2050894 | 2005 | 213 | 213 | F5-5 | 22 | 14 | 27 | Out | 4 | Out |
| 2050806 | 2005 | 213 | 213 | F5-5 | 22 | 14-12 | 26 | In | 4 | Out |
| 2050776 | 2005 | 213 | 213 | F5-5 | 22 | 14 | 16 | Out | 4 | Out |
| 2070054 | 2007 | 213 | 213 | F5-5 | 22 | 14 | 12 | Out | 5 | Out |
| 2061654 | 2006 | 213 | 213 | F1-2 | 22 | 14 | 25 | Out | 4 | Out |
| 2061550 | 2006 | 213 | 213 | F1-33 | 22 | 14 | 9 | Out | 7 | Out |
| 2060937 | 2006 | 213 | 213 | F5-5 | 22 | 14 | 17 | In | 5 | Out |
| 2071759 | 2007 | 213 | 213 | F5-5 | 22 | 14 | 20 | In | 8 | Out |
| 2071290 | 2007 | 213 | 213 | F1-7 | 22 | 14 | 21 | Out | 4 | Out |
| 2071260 | 2007 | 213 | 213 | F5-5 | 22 | 14 | 12 | Out | 4 | Out |
| 2071139 | 2007 | 213 | 213 | F5-5 | 22 | 14 | 30 | Out | 7 | Out |
| 2071109 | 2007 | 213 | 213 | F5-5 | 22 | 14-12 | 10 | Out | 7 | Out |
| 2070736 | 2007 | 213 | 213 | F5-5 | 22 | 14 | 15 | Out | 5 | Out |
| 2081743 | 2008 | 213 | 213 | F5-1 | 22 | 14 | 27 | Out | 7 | Out |
| 2081595 | 2008 | 213 | 213 | F5-1 | 22 | 14 | 20 | In | 4 | Out |
| 2081094 | 2008 | 213 | 213 | F3-3 | 22 | 14 | 15 | Out | 7 | Out |
| 2081064 | 2008 | 213 | 213 | F5-1 | 22 | 14 | 19 | Out | 8 | Out |
| 2080894 | 2008 | 213 | 213 | F5-1 | 22 | 14 | 20 | In | 7 | Out |
| 2080861 | 2008 | 213 | 213 | F5-1 | 22 | 14 | 32 | In | 5 | Out |
| 2080309 | 2008 | 213 | 213 | F5-1 | 22 | 14 | 11 | In | 4 | Out |

^a^ ST, sequence type.

^b^ FetA and PorA are used for molecular typing of meningococci. The variability of PorA is mainly concentrated in two loops called variable region 1 (VR1) and variable region 2 (VR2). The codes of the sequences of VR1 and VR2 are available at http://pubmlst.org/neisseria/PorA/. The codes of the sequence of the variable region of FetA are available at <http://pubmlst.org/neisseria/FetA/>. NA, not available.

^c^ Rn, number of tetranucleotide repeats. Data for *autA* are from [4].

^d^ In or out of frame was defined by the number of repeats. Data for *autA* are from [4].
