# Supplementary material for: Expression of the Gene for Autotransporter AutB of Neisseria meningitidis Affects Biofilm Formation and Epithelial Transmigration
Source: Front Cell Infect Microbiol. 2016 Nov 22;6:162. doi: 10.3389/fcimb.2016.00162 (PMC5118866; doi:10.3389/fcimb.2016.00162)
Supplement: Supplementary file 4 [file Image1.PDF]

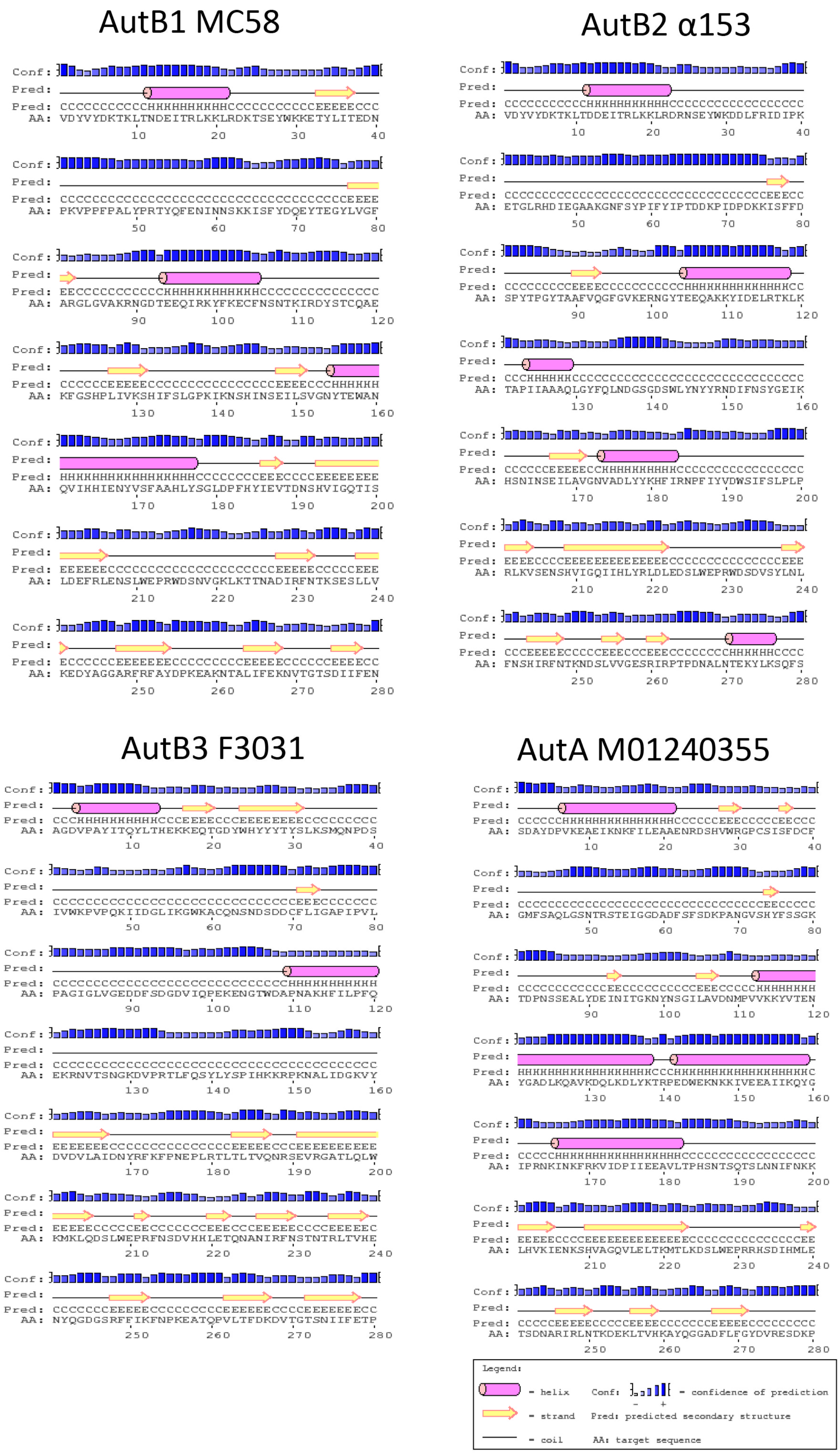

Fig S1. Secondary structure predictions of the N-terminal part of the passengers of AutB variants and AutA.
