# Supplementary material for: Expression of the Gene for Autotransporter AutB of Neisseria meningitidis Affects Biofilm Formation and Epithelial Transmigration
Source: Front Cell Infect Microbiol. 2016 Nov 22;6:162. doi: 10.3389/fcimb.2016.00162 (PMC5118866; doi:10.3389/fcimb.2016.00162)
Supplement: Supplementary file 5 [file Image2.PDF]

```
Protein    Strain    Sequence

                     ──► N-terminal passenger
AutB1      MC58      V--DYVYDKTKLTNDE-------------ITRLKKLRDKTSEYWKKETYLITEDNP----
AutB1      477       V--DYTYDNSKLTNEQ-------------IERLKKLRDRNSEYWKKETYLLKSPPS----
AutB2      α153      V--DYVYDKTKLTDDE-------------ITRLKKLRDRNSEYWKDDLFRIDIPKETGLR
AutB2      Hi375     V--DYVYDKTKLTDDE-------------ITRLKKLRDRNSEYWKDDLFRLDIPKETGLR
AutB3      F3031     V---PAYITQYLTHEKKEQT-GDYWHYYYTYSLKSMQNPDSIVWK------PVPQK--II
AutB3      F3047     VSGEPKYVQEYFVKERGIQYFGEGSGASVFYSLKDMLDRNSVAWS------PVPKQ--II
                     *     *    :..:.                **.: :  *  *.

AutB1      MC58      ----------KVPPFPALYPR--TYQFENINNSKKISFYDQEYTEGY--LVGFARGLG--
AutB1      477       ----------NFPDIPVLFPKDSSVPFENIDNSKAISFYDQKYTEDY--LVGFAQGFG--
AutB2      α153      H-DIEGAAKGNFS-YPIFYI---PTDDKPIDPDKKISFFDSPYTPGY--TAAFVQGFG--
AutB2      Hi375     H-DIKGAATGNFS-YPVIGS---YYNDKPIDPDKKISFFDSPYTPGY--TAAFVQGFG--
AutB3      F3031     DGLIKGWKACQNS--------------NDSDDCF--LIGAPIPVLPAGIGLVGEDDFSDG
AutB3      F3047     D-YITNTEACYFS-HP----------DSPKDDCQ--LFGSNTYEKKWFLTDFSEQKYG--
                                 .              .  :      :                   .

AutB1      MC58      ----VAKRNGDTEEQIRKYFKECFNSNTKIRDYS---------TCQAEKFGSHPLIVKSH
AutB1      477       ----VAKRNGDTEEPVRKYFKECLNTGNYNND-----------NCKANPLAS-SVSIKSD
AutB2      α153      ----VKERNGYTEEQAKKYIDE-LRTKLKTAPIIAAAQLGYFQLNDGSGDSWLYNYYRND
AutB2      Hi375     ----VKERNGNTEEQAKQFIDE-FRTRLKKGTYFHS--LGFF-YSDGMPYTWLYEFYKND
AutB3      F3031     DVIQPEKENGTWDAPNAKHFILPFQEKRNV-------------TSNGKDVPR--TLFQSY
AutB3      F3047     ----AEEENGIFGNEYAKHFVYPFREDIPF-------------YSESEKLYKLPVLYQSY
                           :.**       :.:   :.                    ..          :.

AutB1      MC58      IFSLGPKI-----KNSHINSEILSVGNYT-EWANQVIHHIE--NYVSFAAHLYSGLDPFH
AutB1      477       IFTPRPTI-----KNSHINSEILAVGNYT-EL---MLAAQP--SASSWAEHLYS--DPGL
AutB2      α153      IFNSYGEI-----KHSNINSEILAVGNVA-DLYYKHFIRNPF-IYVDWS--IFS--LPLP
AutB2      Hi375     IFNSYKVV-----KHSNINSEILAVGNVA-DLYYKHYIRSPIPLYADWS--ILT--LPLP
AutB3      F3031     LYSPIHKK---RPKNALIDGKVYDVDVLAIDNYRFKFPNEP---------------LRTL
AutB3      F3047     LYDNYETEKTSRPLNALINNRVYDFNVLAIDNYRSKFPDE----------------TRGL
                     ::            :: *:..:  ..  : :
                                               N-terminal passenger ◄─ ─► Linker
AutB1      MC58      YIEVTDNSHVIGQTISLDEFRLENSLWEPRWDSNVGKLKTTNADIRFNTKSESLLVKED-
AutB1      477       SLTVQDNSHVIGQTIDLGFLQLTNSLWEPRWNSNIDYLVTENAEIRFNTKNESLLVEGY-
AutB2      α153      RLKVSENSHVIGQIIHLYRLDLEDSLWEPRWDSDVSYLNLFNSHIRFNTKNDSLVVGESR
AutB2      Hi375     RLKVSENSHVIGQIIHLYRVDLENSLWEPRWDSDVSYLNLNNGHIRFNTKNDSLVVGESR
AutB3      F3031     TLTVQNRSEVRGATLQLWKMKLQDSLWEPRFNSDVHHLETQNANIRFNSTNTRLTVHEN-
AutB3      F3047     TLTVKNQSEVRGATLQLLKMVLQDSLWEPRFNSDVHHLETQNANIRFNSTNTRLTVHEN-
                      : * :.*.* *  : *  . * :******::*::  *   *..****:..  * *

AutB1      MC58      ---------------------------------------YAG-GARFRFAYDPKE--AKN
AutB1      477       ---------------------------------------YAG-GARFRFAYDLKDKAPET
AutB2      α153      IRPTPDNALNTEKYLKSQFSDTGYHS--------SVRSTYIG-YPSIRFAYDLSEREADK
AutB2      Hi375     IRPTPDNALLPEKSLKREGNDIGYHSNSEYIYQNSSEDHYAD-YSSIHFAYDLSEREADK
AutB3      F3031     ---------------------------------------YQGDGSRFFIKFNPKE--ATQ
AutB3      F3047     ---------------------------------------YQGDGSRFFIKFNPKE--ATQ
                                                            * .  . : : :: .:  .

AutB1      MC58      TALIFEKNVTGTSDIIF-ENPIDDLKSLDGHQIIKVNGTADKHAFRLSGKHQKGIYTLSL
AutB1      477       PVLTFEQNITGTSDIIF-ENPIDDLKSLDGHQIVKVNGTADKNAFRLSGKYRKGIYTLSL
AutB2      α153      PVLTLKSKVTGKTAIVFEEKALNNLKNLTYRQLIKTETDVEPDAFFLLEEYKKGRYRLFL
AutB2      Hi375     PVLTLKSKVTGKTAIVFEEKALNNLKNLTYRQLIKTETDVEPNAFYLLEEYKKGRYRLFL
AutB3      F3031     PVLTFDKDVTGTSNIIF-ETPIEDLKSLDGHQIIKVNGTADKRAFRLSGKHQKGIYNLFL
AutB3      F3047     PVLTFDKDVTGTSNIVF-EKPIDDLKSLDGHQIIKVNGTADKHAFRLSGKHQKGIYTLSL
                     ..* :...:**.: *:* *..:::**.*  :*::*.:  .:  ** *  :::** * * *
                                 Linker ◄─ ─►     α-helix      ◄─ ─► β-barrel
AutB1      MC58      QQRPEGFFTKVQE----RDDMAIYAQQAQAANTLFALRLNNKNSDIFDRTLPRKGLWLRV
AutB1      477       QQRLEGFFTKLQE----RDDIAIYAQQAQAANTLFALRLNDKNSDIFDRTLPRKGLWLRV
AutB2      α153      RQCPNGFCIGVEKLAIPTHLVASYAQQAQAANTLFNLRLNDKNSDIFDRTLPRKGLWLRV
AutB2      Hi375     QQCPNGFCIGVEKLAIPTHLVASYAQQAQAANTLFALRLNDKNSDIFDRTLPRKGLWLRL
AutB3      F3031     KQCQGGFCTGIQE----RNDIAIYAQQAQAANTLFALRLNDKNSDIFDRTLPRKGLWLRL
AutB3      F3047     QQRPEGFFTKVQE----RDDIAIYAQQAQAANTLFALRLNDKNSDIFDRTLPRKGLWLRL
                     :*   **   :::     .  :* ************ ****:******************:

AutB1      MC58      IDGHSNQWVQGKTAPVEGYRKGVQLGGEVFTWQNESNQLSIGLMGGQAEQRSTFHNPDTD
AutB1      477       IDGHSSQWVQGKTAPLEGYRKGVQLGGEVFTWQNESNQFSVGLMGGQAEQRSTFRNPDTD
AutB2      α153      IDGHSNQWVQGKTAPVEGYRKGVQLGGEVFTWQNESNQLSVGLMSGQAEQRSTFRNPDTD
AutB2      Hi375     ISGHLSQDVQGKTAPVEGNRKGIQLGGDVFSLQNQDYQFSFGLMGGQAEQRSTFRNPDTG
AutB3      F3031     ISGHLSQDVQGKTAPVEGNRKGIQLGGDVFSLQNQDYQLSFGLMGGQAEQRSTFRNPDTD
AutB3      F3047     ISGHLSQDVQGKTAPVEGNRKGIQLGGDVFSLQNQDYQLSFGLMGGQAEQRSTFRNSDTD
                     *.** .* *******:** ***:****:**: **:. *:*.***.*********:*.**.

AutB1      MC58      NLTTGNVKGFGAGVYATWHQLQDKQTGAYADSWMQYQRFRHRINTEDGTERFTSKGITAS
AutB1      477       NLTTGNMKGLGAGVYATWHQLQDKQTGAYVDSWAQYQRFRHRINTEDGTERFTSKGITAS
AutB2      α153      NLTTGNVKGFGAGVYATWHQLQDKQTGAYADSWVQYQRFRHRINTEDATERFTSKGITAS
AutB2      Hi375     NVTTGSMKGFGAGVYVTWHQLQDKQTGAYIDSWVQYQRFRHRINTEDGTESFTSKGITAS
AutB3      F3031     NLTTGNMKGFGAGIYATWHQLQDKQTGAYVDSWMQYQRFRHNVNSEDGTERFTSKGITAS
AutB3      F3047     NLTTGSMKGFGAGIYATWHQLQDKQTGAYVDSWVQYQRFRHRINTEDGIERFTSKGITAS
                     *:***.:**:***:*.************* *** *******.:*:**. * *********
```

```
AutB1   MC58    IEAGYNALLAEHFTKKGNSLRVYLQPQAQLTYLGVNGKFSDSENAHVNLLGSRQLQTRVG
AutB1   477     IEAGYNALLAEHLTGKGTQIRFYLQPQAQLTYLGVNGKFSDSENSQVNLLGSRQLQSRVG
AutB2   α153    IEAGYNALLAEHFTKKGNSLRVYLQPQAQLTYLGVNGKFSDSENAHVNLLGSRQLQTRVG
AutB2   Hi375   IEAGYNALLAEHLTGKGTQIRFYLQPQAQLTYLGVNGGLTDSGNSKVNLLGSRQLQSRVG
AutB3   F3031   IEAGYNALLAEHVTRKGTQIRFYLQPQAQLTYLGVNGGLTDSGDSKVNLLGSRQLQSRVG
AutB3   F3047   IEAGYNALLAEHLTEKGTQIRFYLQPQAQLTYLGVNGDLTDSGNSKVNLLGSRQLQSRVG
                ************.* **..:*.*************** ::** :::**********:***

AutB1   MC58    VQAKAQFSLYKNIAIEPFAAVNALYHNKPFGVEMDGERRVINNKTAIESQLGVAVKIKSH
AutB1   477     VQAKAQFLLNKNIVIQPFAAVNTLYHSKPFGVEIDGERRVINNKTAIESQFGIAVKIKSH
AutB2   α153    VQAKAQFSLYKNIAIEPFAAVNALYHNKPFGVEIDGERRVINNKTAIESQLGVAVKIKSH
AutB2   Hi375   VQAKAQFLLNKNIVIQPFAAVNTLYHSKPFGVEIDGERRVINNKTAIESQFGIAVKIKSH
AutB3   F3031   IQAKAQFLLNKNIVIQPFVAVNALYHSKPFGVEMDGERRVINNKVAIESQLGVALKIKSH
AutB3   F3047   AQAKAQFTFTNGVIFQPFVALNSIYQQKPFGVEIDNERRVINNKTAIESQLGVALKIKSH
                 ****** :  :.:  ::**.*:*::*:.******:*.********.*****:*:*:*****

AutB1   MC58    LTLQATFNRQTGKHHQAKQGALNLQWTF
AutB1   477     LTLQATFNRQTGKHHHAKQGALNLQWTF
AutB2   α153    LTLQATFNRQTGKHHQAKQGALNLQWTF
AutB2   Hi375   LTLQATFNRQTGKHHHAKQGALNLQWTF
AutB3   F3031   LTLQATFNRQTGKHHHAKQGVLNLQWTF
AutB3   F3047   LTLQATFNRQTGKHHHAKQGALNLQWTF
                ***************:****.*******
```

Fig S2. Alignment of the predicted mature AutB proteins of various *N. meningitidis* and *H. influenzae* strains. AutB proteins are classified as B1, B2, or B3 based on clustering of the passenger domain (Fig. 2). Asterisks, colons, and periods below the sequences indicate identical amino acids in all aligned sequences, and positions with conserved and semi-conserved substitutions, respectively. The cysteines in the passenger domains are highlighted. The sequence of AutB of strain H44/76 is identical to that of strain MC58. The shadow indicates the region of the AutB of H44/76 that was used to raise anti-AutB1 antiserum and sequence differences with other AutB variants. The sequences for the N-terminal part of the passenger, the linker, the α-helix and β-barrel domain are indicated with arrows.
