# Supplementary material for: Expression of the Gene for Autotransporter AutB of Neisseria meningitidis Affects Biofilm Formation and Epithelial Transmigration
Source: Front Cell Infect Microbiol. 2016 Nov 22;6:162. doi: 10.3389/fcimb.2016.00162 (PMC5118866; doi:10.3389/fcimb.2016.00162)
Supplement: Supplementary file 6 [file Image3.PDF]

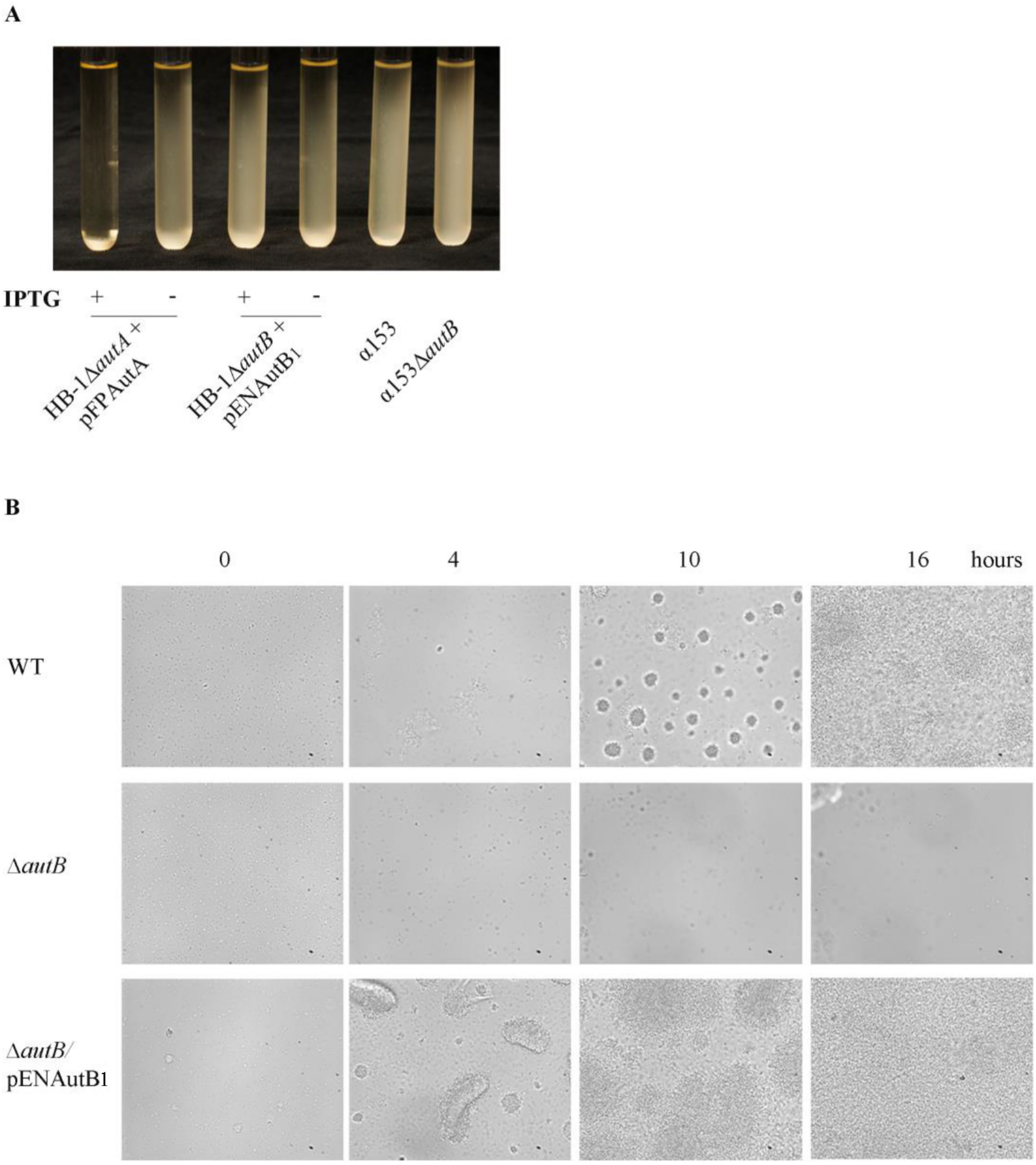

Fig S3. Settling assays and biofilm development.

(A) Cultures of HB-1Δ*autA* carrying pFPAutA, HB-1Δ*autB* carrying pENAutB1, α153 and α153Δ*autB* were grown overnight with or without IPTG as indicated, adjusted to the same OD550, left standing for 2 h and then photographed. Settling of the cells because of autoaggregation was only observed in the culture of strain HB-1Δ*autA* carrying pFPAutA after growth with IPTG.
(B) Biofilm development of α153 and α153Δ*autB* and α153Δ*autB* carrying pENAutB1 grown with IPTG during 16 h under flow conditions. Bacteria were inoculated into the chamber to initiate biofilm formation, and after 1 h the flow was started. Microscopy pictures were taken immediately after flow initiation (t=0) and at different time points, as indicated.
